# Latent-Space Modeling Reveals Transcriptional Strategy Divergence in Flax Root Drought Response

**DOI:** 10.3390/plants15213494

**Published:** 2026-11-09

**Authors:** Lili Guo, Gongfu Shi, Yao Wang, Rui Xie, Shuli Wei, Xinhui Huang, Huimin Shi, Jing Fang, Ying Liu, Jie Ma, Xiaoqing Zhao

**Affiliations:** 1 Key Laboratory of Black Soil Protection and Utilization, State Key Laboratory of Agricultural Ecology in the Agro-Pastoral Ecotone (Cultivation), Inner Mongolia Academy of Agricultural & Animal Husbandry Sciences, Ministry of Agriculture and Rural Areas, Hohhot 010031, China; 13474912747@163.com (L.G.); y214575@126.com (G.S.); 13722132892@163.com (Y.W.); xierui12358@163.com (R.X.); wslweishuli@163.com (S.W.); hxhxinhui159@163.com (X.H.); nmgnongda@163.com (H.S.); liuying836354822@126.com (Y.L.); majie19952021@163.com (J.M.); 2 School of Life Science, Inner Mongolia University, Hohhot 010020, China; 3 Agricultural College, Inner Mongolia Agricultural University, Hohhot 010019, China; fangjing721@126.com

**Keywords:** root drought tolerance, WGCNA, β–VAE, SHAP, flax (*Linum usitatissimum* L.)

## Abstract

Drought stress severely constrains flax (*Linum usitatissimum* L.) production, yet the transcriptional logic underlying genotypic variation in root drought tolerance remains unclear. We subjected 20 flax cultivars to drought at peak flowering, constructed a continuous Drought Tolerance Index (DTI) based on biomass maintenance, and generated 120 root transcriptome profiles. Integrative analyses combining PCA/UMAP, β–variational autoencoder (β–VAE) latent space modeling, gene co-expression-network analysis (WGCNA), Random Forest–SHAP (SHapley Additive exPlanations), and virtual knockout were performed. We found that drought-tolerant and drought-sensitive cultivars underwent transcriptional remodeling of similar magnitude, but diverged in direction along a latent strategy axis, corresponding to “water-conserving” and “growth-maintenance” strategies, respectively. The water-conserving strategy was characterized by upregulation of genes involved in stress protection, protein folding, and ABA signaling, whereas the growth-maintenance strategy showed elevated expression of oxidative and cell wall-related genes. Drought strengthened co-expression coordination within a core regulatory module—enriched for transcription factors and hormone signaling—despite its overall expression decline. A small set of leverage genes were identified that accurately predicted DTI; these genes were not network hubs, yet their virtual knockout induced substantial drift in the latent strategy space, confirming their non-redundant predictive information. This framework uncovers transcriptional strategy divergence underlying flax root drought tolerance and offers candidate targets for molecular breeding.

## 1. Introduction

Drought is the single most devastating abiotic stress limiting global crop production. The frequency, duration, and intensity of drought episodes continue to rise under climate change, threatening food and industrial crop security worldwide [1,2]. Unlike many abiotic stresses, drought exerts damage across virtually every stage of plant development, with the reproductive phase being particularly vulnerable: yield losses of 30–50% are commonly reported even in major cereals under terminal drought [3,4].

Flax (*Linum usitatissimum* L.) is an ancient multipurpose crop cultivated globally for its fiber and omega-3-rich seeds [5,6]. Over the past decade, significant genomic resources have been developed for flax, including a high-quality reference genome [7], chromosome-scale pseudomolecules [8], and, most recently, a gapless telomere-to-telomere assembly of 482.51 Mb [9]. These advances provide an unprecedented foundation for functional genomic dissection of complex traits. Yet despite this progress, flax production in rainfed and semi-arid environments remains severely constrained by drought [10,11]. Substantial genotypic variation in drought tolerance exists across flax germplasm collections. A recent comprehensive evaluation of 243 flax accessions classified them into five drought-tolerance classes, ranging from drought-tolerant to drought-sensitive, with oil-type flax generally outperforming fiber-type under water deficit [12]. However, the molecular mechanisms that underpin this variation remain largely unknown.

The root system constitutes the plant’s frontline organ for drought adaptation. Root system architecture—including rooting depth, branching angle, and lateral root density—directly determines the plant’s capacity to extract water from deeper soil layers [13,14,15]. The paradigm established by the cloning of DEEPER ROOTING 1 (DRO1) in rice demonstrated that a single genetic locus controlling root growth angle can significantly enhance deep rooting and confer drought avoidance under field conditions [14,16]. In flax, recent genome-wide association studies have identified over 80 quantitative trait loci (QTL) associated with root morphological traits under drought stress, explaining 49–73% of phenotypic variation [17]. These studies provide compelling evidence that root architectural variation in flax is under genetic control, but they largely stop at the level of QTL intervals—the downstream transcriptional programs that translate genotype into root drought response remain to be systematically delineated.

At the molecular level, water deficit triggers a complex regulatory cascade centered on the phytohormone abscisic acid (ABA). ABA signaling activates a suite of transcription factors—including members of the MYB, NAC, bZIP, AP2/EREBP, and HD-ZIP families—that orchestrate stomatal closure, osmotic adjustment, and root architectural remodeling [18,19]. ABA also engages extensive crosstalk with auxin signaling to modulate root growth direction and branching under stress. While transcriptomic profiling under drought has been conducted in individual flax genotypes, revealing thousands of differentially expressed genes involved in photosynthesis, reactive oxygen species detoxification, and osmolyte biosynthesis [13,20], these studies capture only single-genotype responses under controlled conditions and cannot reveal the divergence in regulatory strategies that exists across genetically diverse germplasm.

Critically, the question of how different flax genotypes deploy distinct or shared transcriptional strategies in response to field drought remains unanswered. Addressing this question requires more than pairwise differential expression analysis: it demands a framework that can (i) identify core transcriptional modules conserved across genotypes as universal drought-response programs, (ii) capture genotype-specific network perturbations that distinguish tolerant from susceptible accessions, and (iii) map the continuum of transcriptional strategies across a diversity panel onto a low-dimensional landscape where biological “strategy space” can be quantitatively visualized. Weighted gene co-expression network analysis (WGCNA) provides a powerful systems-biology tool for identifying expression modules correlated with phenotypic traits [21,22], and its integration with machine learning-based feature attribution—such as Random Forest combined with SHapley Additive exPlanations (SHAP) [23,24]—enables unbiased prioritization of the most impactful regulatory nodes within complex networks. Complementing these approaches, variational autoencoders (VAEs) offer an unsupervised framework to learn continuous, nonlinear latent representations (a low-dimensional coordinate system where each point represents the global transcriptomic state of a sample) of high-dimensional transcriptomic data [25]. This provides a principled means to interrogate the “strategy space” across genotypes.

Here, we address these gaps through an integrative field-based root transcriptomic study of 20 flax genotypes encompassing a broad spectrum of drought responses, ranging from drought-tolerant to drought-sensitive genotypes. We imposed a 30-day water-withholding treatment at the full flowering stage—a phenological window of high drought sensitivity—and quantified shoot growth and biomass maintenance to construct a composite Drought Tolerance Index (DTI). Leveraging a multi-layered computational framework that combines PCA/UMAP for global expression overview, β-VAE for latent strategy space modeling, WGCNA for co-expression network analysis, and Random Forest–SHAP for interpretable gene prioritization, we characterized the transcriptional landscape of root drought response at complementary levels. This approach allowed us to determine whether genotypic variation in drought resilience is associated with the direction or the magnitude of transcriptional reprogramming, to identify network modules that undergo stress-induced topological reorganization, and to pinpoint individual genes that most accurately predict DTI from peripheral network positions. By integrating these analytical layers, we uncover a hierarchical regulatory architecture in which drought adaptation is governed by strategy direction rather than response intensity, and in which predictive “lever genes” operate at the network periphery rather than as central hubs. These findings provide a conceptual framework for dissecting drought adaptation strategies in flax and offer candidate targets for molecular breeding of climate-resilient cultivars.

## 2. Results

### 2.1. Widespread Growth Inhibition in Flax Varieties Under Drought Stress

**Figure 1:**
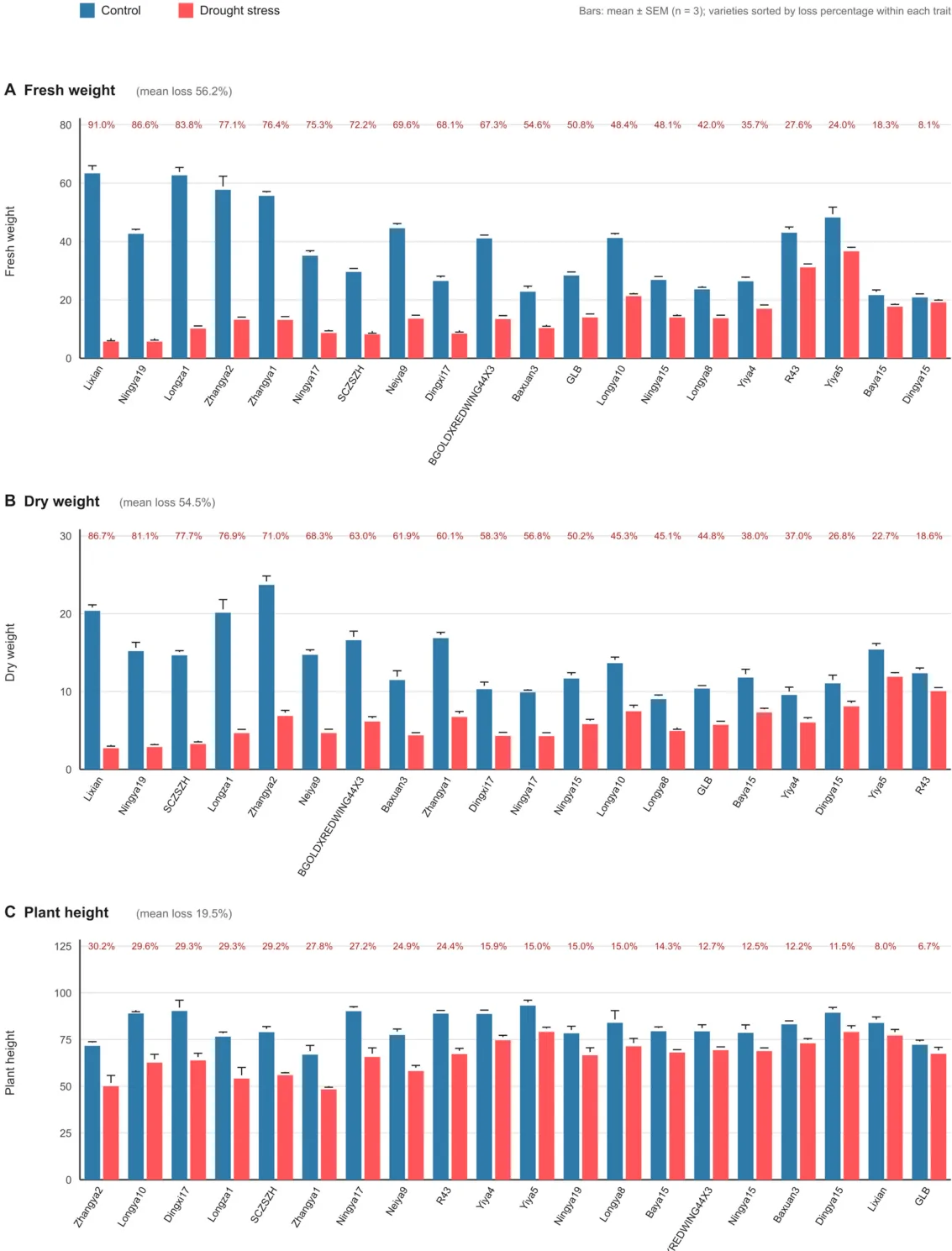
Effects of drought stress on agronomic traits across 20 flax varieties. Bar charts showing Fresh weight (**A**), Dry weight (**B**), and Plant height (**C**) under control (blue) and drought stress (red) conditions. Bars represent mean ± SEM (*n* = 3). Varieties within each panel are ordered by decreasing loss percentage. The percentages above the bars indicate the specific loss percentage induced by drought stress for each variety.

Compared with the well-watered controls, drought stress caused highly significant reductions in all three measured traits across the 20 varieties (*p* < 0.001; Figure 1). Across all varieties, biomass traits (fresh weight and dry weight) showed average losses of 56.2% and 54.5%, respectively, whereas plant height was considerably less affected, with a mean reduction of only 19.5% (Figure 1). However, striking differences in absolute performance were observed among genotypes under stress. For instance, the most severely affected variety, Lixian, lost only 91% of its control fresh weight under drought (63.34 g vs. 5.70 g) and 86.7% of its control dry weight (20.38 g vs. 2.72 g). In stark contrast, the least affected variety in terms of fresh weight, Dingya15, showed a loss of only 8.1% (20.83 g vs. 19.16 g), while R43 exhibited the smallest loss in dry weight (18.6%, 12.36 g vs. 10.06 g). Plant height also exhibited genotypic variation, but the absolute range under drought was relatively narrow, with most varieties maintaining stature above 50 cm. These results indicate that while drought imposes a widespread and statistically significant inhibitory effect, the magnitude of response is highly trait-dependent and genotype-specific, with biomass parameters showing the greatest vulnerability.

**Figure 2:**
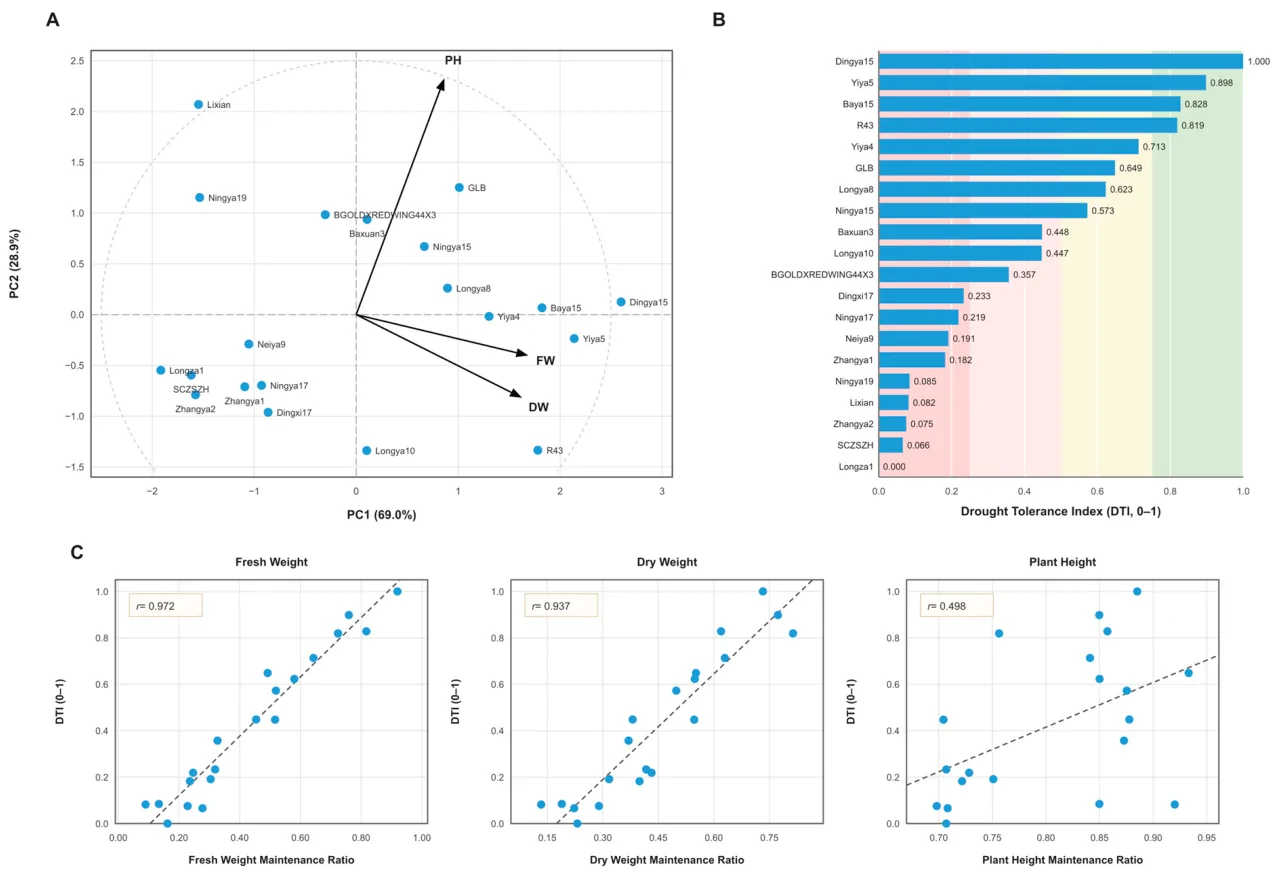
Drought tolerance evaluation based on trait maintenance ratios across 20 flax varieties. (**A**) PCA biplot of Z-standardized ratios (PC1: 69.0%, PC2: 28.9%). Arrows show trait loadings (FW, DW, PH). (**B**) Drought Tolerance Index (DTI) derived from PC1 and scaled 0–1, with background shading from green to red denoting four tolerance classes (from highly tolerant to highly sensitive). (**C**) Pearson correlations (*r*) between DTI and individual trait ratios.

To integrate the three trait responses into a comprehensive metric, we calculated trait-specific maintenance ratios (drought/control) and subjected them to principal component analysis (PCA), yielding a composite Drought Tolerance Index (DTI). PCA of the standardized ratios extracted two principal components explaining 97.9% of the total variance, with PC1 (69.0%) receiving strong positive loadings from all three traits Figure 2A, particularly from fresh weight (0.676) and dry weight (0.651). We therefore adopted PC1 as DTI and scaled it to a 0–1 range. Based on DTI, the 20 varieties were arranged along a continuous tolerance gradient, with Dingya15 showing the highest tolerance (DTI = 1.000) and Longza1 the most severe susceptibility (DTI = 0.000); the remaining varieties fell sequentially between these two endpoints in descending DTI order (Figure 2B). Correlation analyses further validated DTI as a biologically meaningful metric: DTI was tightly correlated with fresh-weight (*r* = 0.972) and dry-weight (*r* = 0.937) maintenance ratios, but only weakly with plant-height maintenance (*r* = 0.498), confirming that biomass retention capacity is the primary driver of DTI (Figure 2C).

### 2.2. Prolonged Drought Stress Reshapes the Root Transcriptome Landscape

**Figure 3:**
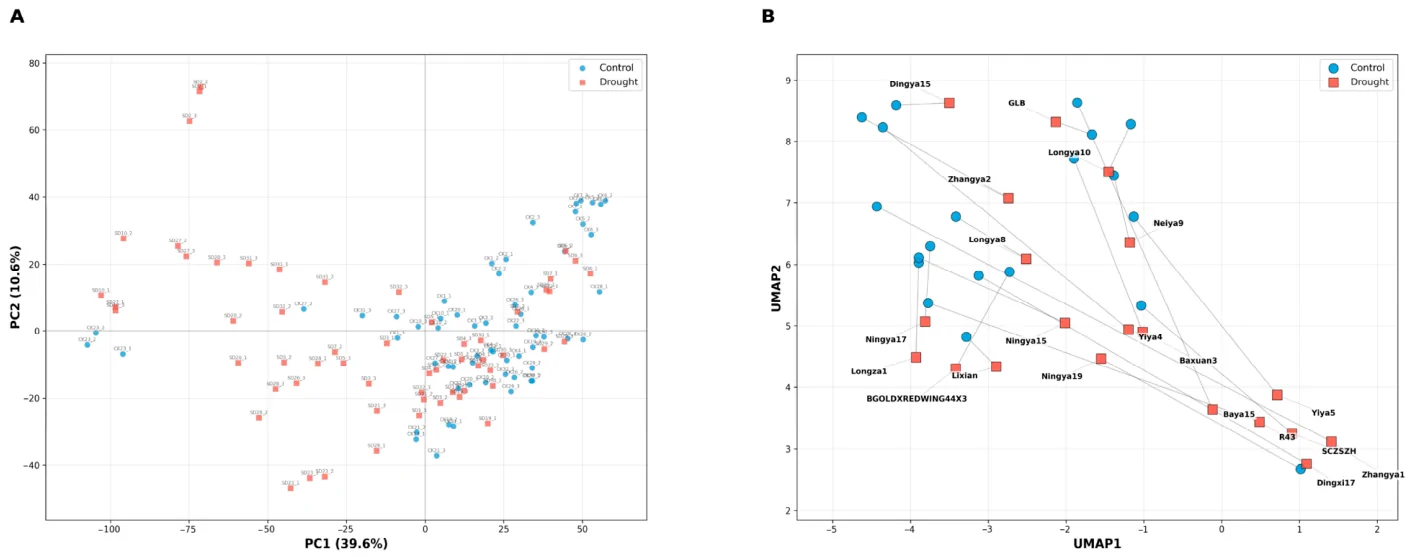
PCA and UMAP of 120 flax transcriptomes. (**A**) PCA of all samples colored by treatment (blue: control; red: drought). PC1/PC2 (39.6%/10.6%) capture treatment and genotype effects. (**B**) UMAP of varietal means, connecting control and drought for each variety.

To obtain an unbiased overview of the global transcriptomic structure, we first performed PCA and UMAP on the 4398 high-variance genes across all 120 samples (20 varieties × 2 treatments × 3 replicates). PCA revealed that drought treatment is the dominant source of variation, with PC1 explaining 39.6% of the total variance and cleanly separating control and drought-treated samples (Figure 3A). PC2 (10.6% of variance) primarily captured inter-varietal transcriptional differences within each treatment. Uniform Manifold Approximation and Projection (UMAP) was subsequently applied to resolve cultivar-level response trajectories. At the cultivar mean level, most varieties shifted downward along UMAP2 from control to drought, while a few, such as GLB and Ningya19, displayed opposing or near-zero displacements, indicating variety-specific response strategies (Figure 3B).

### 2.3. β-VAE Latent Space Reveals Two Distinct Drought Adaptation Strategies

**Figure 4:**
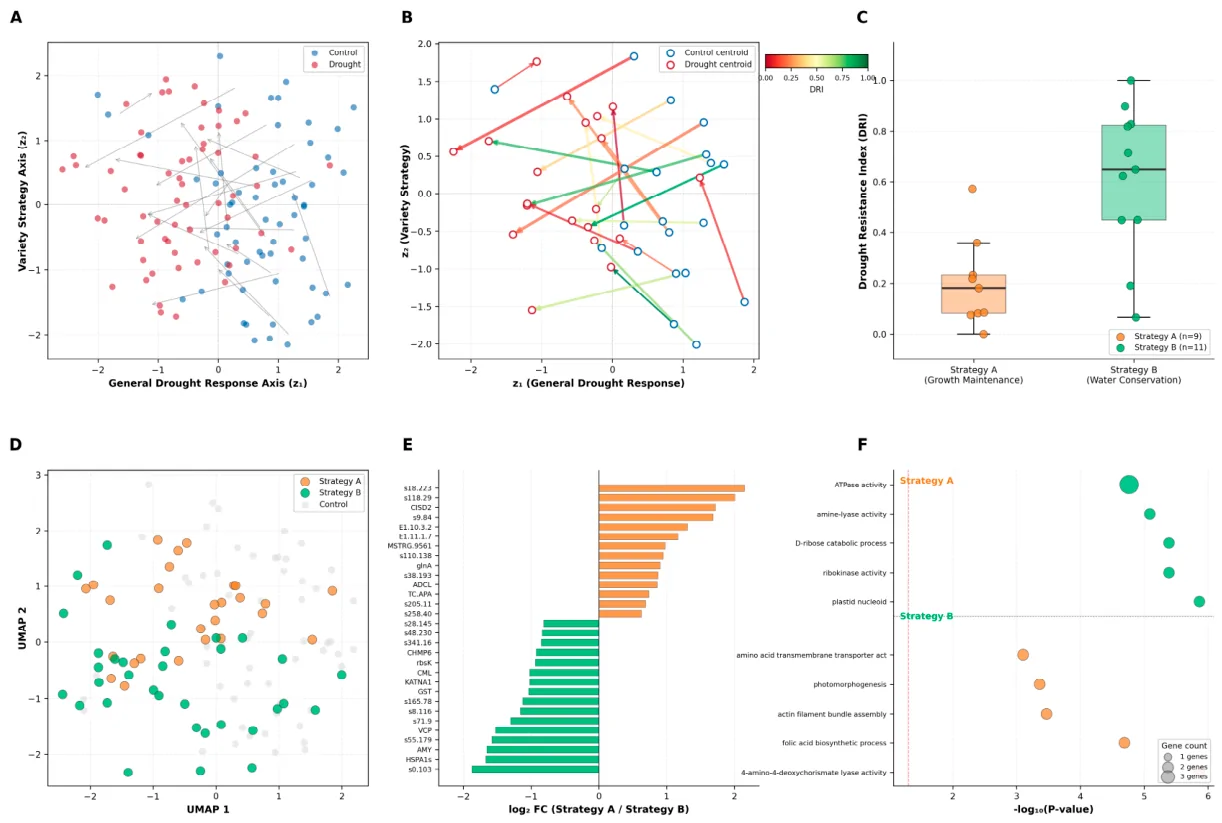
β-VAE latent space reveals two distinct drought adaptation strategies in flax. (**A**) 2D projection of the 8-dimensional latent space (z_1_–z_2_); samples colored by treatment. z_1_ separates Control from Drought; z_2_ captures inter-variety variation. The grey lines connect the Control and Drought centroids for each variety, representing their adaptation trajectories. (**B**) Adaptation trajectories for 20 varieties (Control toDrought centroids) in the z_1_–z_2_ plane. (**C**) K-means clustering (k = 2) on the PLS strategy axis coordinates of drought centroids, defining Strategy A (Growth Maintenance) and Strategy B (Water Conservation). DTI difference, *p* = 7.84 × 10^−3^ (Welch’s *t*-test). Boxes show the median and interquartile range (IQR); whiskers extend to 1.5 × IQR. (**D**) UMAP validation of strategy separation using the top 500 variable genes. (**E**) Differential expression (log2 fold-change) of the top 30 decoded genes between Strategy A and Strategy B. The *Y*-axis lists the gene IDs. Orange bars indicate genes up-regulated in Strategy A (positive log2FC), while green bars indicate genes up-regulated in Strategy B (negative log2FC). (**F**) GO enrichment of decoded genes; top terms for each strategy are shown (FDR < 0.05).

Having confirmed treatment robustness and inter-variety heterogeneity, we next asked whether the global transcriptional response could be compressed into a low-dimensional “strategy space” where the direction, rather than the magnitude, of transcriptomic change distinguishes tolerant from susceptible genotypes. We trained a β-VAE (β = 4, 8 latent dimensions) on the full 120-sample expression matrix. In the projected space, Control and Drought samples were fully separated along the first axis (z_1_, “General Drought Response Axis”), whereas the orthogonal second axis (z_2_, “Variety Strategy Axis”) captured substantial inter-genotype divergence among drought samples and correlated significantly with DTI (PLS *r* = −0.586, *p* < 0.01; Figure 4A). Connecting each variety’s Control and Drought centroids revealed 20 adaptation trajectories that all pointed toward the drought region yet diverged strongly in their z_2_ components (Figure 4B). Trajectory length did not differ between resistant and sensitive varieties (mean 1.846 vs. 1.847, *p* = 0.604), showing that the amount of transcriptomic remodeling is not predictive of outcome—the direction along the strategy axis is what matters.

Unsupervised K-means clustering on the drought-centroid z_2_ coordinates partitioned the varieties into two strategies: Strategy A (Growth Maintenance, *n* = 9, mean DTI = 0.201) and Strategy B (Water Conservation, *n* = 11, mean DTI = 0.608; Welch’s *t*-test *p* = 7.8 × 10^−3^; Figure 4C). Independent UMAP analysis of the top 500 most variable genes confirmed clear separation between the two groups, ruling out embedding artifacts (Figure 4D). To identify the genes driving the separation of these two strategies in the latent space, we decoded the coordinates of the latent regions corresponding to each strategy back into the original gene expression space. This identified 50 differentially expressed genes (Figure 4E, Table S1): Strategy A (low-DTI, sensitive genotypes) was marked by elevated expression of laccase (E1.10.3.2), peroxidase (E1.11.1.7), glnA (GLUL), ADCL, and CISD2, whereas Strategy B (high-DTI, resistant genotypes) was enriched for AMY (amyA/malS), CML, CHMP6/VPS20, GST, HSPA1s, KATNA1, VCP/CDC48, HSP20, and SNRK2. Functional enrichment further distinguished the strategies (Figure 4F): Strategy A genes were overrepresented in actin filament bundle assembly, amino acid transmembrane transport, folic acid biosynthetic process, and phenylalanine metabolism, as well as in KEGG pathways for phenylalanine/tyrosine/tryptophan biosynthesis, ubiquinone and terpenoid-quinone biosynthesis, and plant–pathogen interaction—consistent with sustained metabolic activity and cell elongation but lacking dedicated stress-protective machinery. In contrast, Strategy B genes were enriched in protein refolding, ATPase activity, ER-associated protein catabolic process, response to heat, glutathione metabolic process, and pyridoxine biosynthetic process, with KEGG pathways for protein processing in the endoplasmic reticulum, circadian rhythm, and vitamin B6 metabolism, indicating a shift toward chaperone-mediated protein quality control, detoxification, and metabolic reprogramming for survival (Table S2). Collectively, the β-VAE latent landscape reveals that flax genotypes employ two fundamentally different drought adaptation strategies: a growth maintenance program prioritizing root proliferation and metabolic activity at the cost of higher water consumption, and a water conservation program emphasizing stress protection and survival. The equivalent magnitude of transcriptomic remodeling across strategies underscores that drought tolerance is governed by the specific identity of rewired gene modules, not by the overall intensity of transcriptional change.

### 2.4. Drought Stress Reshapes Module Co-Expression Network Topology

**Figure 5:**
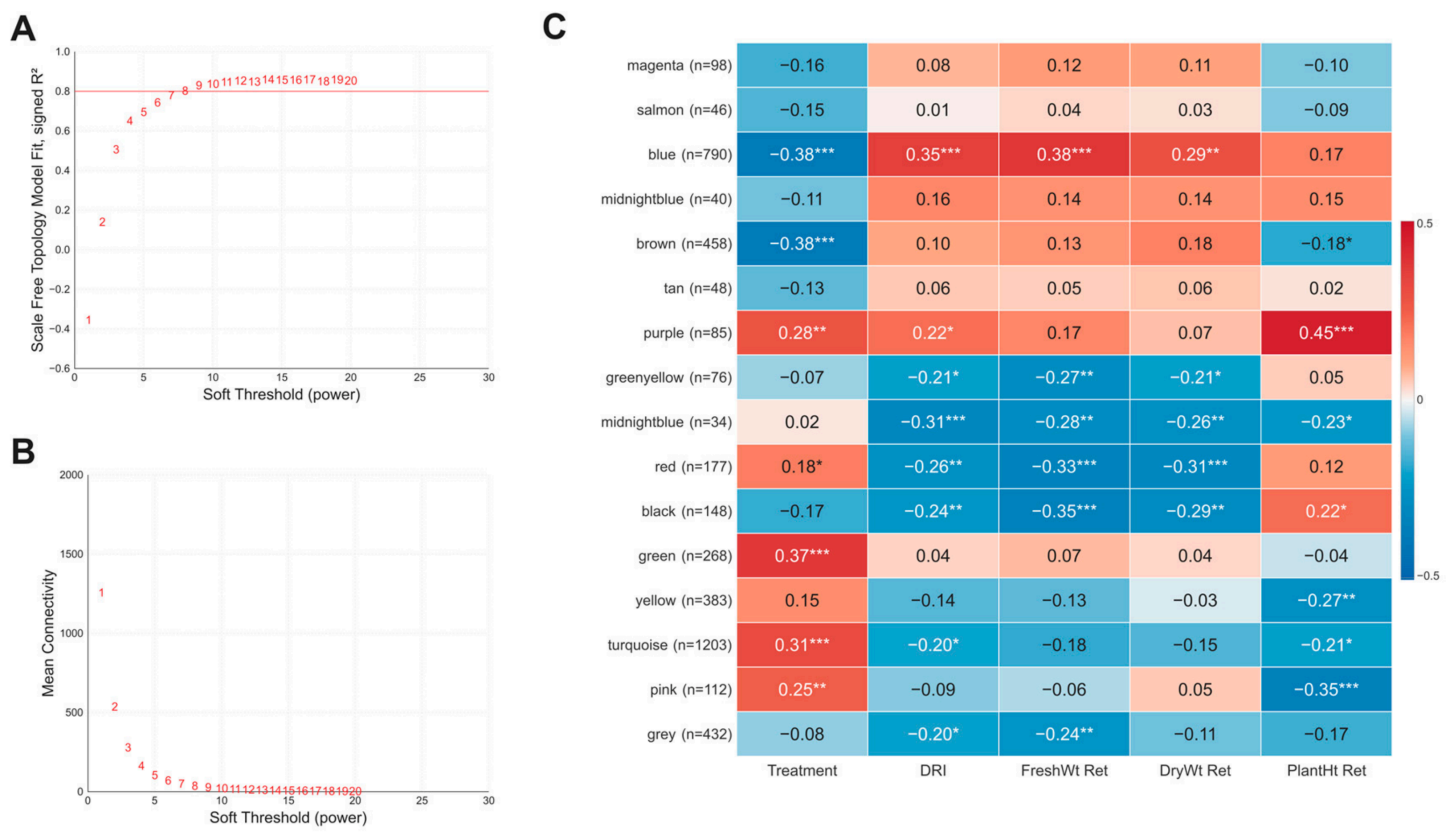
Soft threshold selection and module-trait relationships in weighted gene co-expression network analysis. (**A**) Scale-free topology fit index (R^2^) as a function of soft thresholding power (β). (**B**) Mean connectivity as a function of β. (**C**) Heatmap of correlations between 15 co-expression modules and five traits. Asterisks denote significance levels: * *p* < 0.05, ** *p* < 0.01, *** *p* < 0.001.

To characterize the co-expression architecture underlying the observed strategy divergence, we performed WGCNA on the 4398 high-variance genes. A soft threshold of β = 8 was selected (scale-free R^2^ = 0.803; mean connectivity = 37.1; Figure 5A,B), yielding 15 co-expression modules. The three largest—turquoise (1203 genes), blue (790), and brown (458)—accounted for 55.6% of all clustered genes. Module–trait associations revealed functionally distinct drought-response modules (Figure 5C). The blue module was negatively correlated with drought treatment (*r* = −0.385, *p* = 1.4 × 10^−5^) but positively associated with DTI (*r* = +0.352, *p* = 8.3 × 10^−5^), fresh weight retention (*r* = +0.377, *p* = 2.2 × 10^−5^), and dry weight retention (*r* = +0.294, *p* = 1.1 × 10^−3^), indicating that its down-regulation under drought accompanies higher resilience. In contrast, the green (*r* = +0.371, *p* = 3.0 × 10^−5^) and turquoise (*r* = +0.314, *p* = 4.8 × 10^−4^) modules were drought-induced; the turquoise module additionally showed a negative association with DTI (*r* = −0.200, *p* = 0.028). The purple module, though moderately drought-induced (*r* = +0.284, *p* = 1.6 × 10^−3^), showed the strongest trait correlation with plant height retention (*r* = +0.448, *p* = 2.8 × 10^−7^).

**Figure 6:**
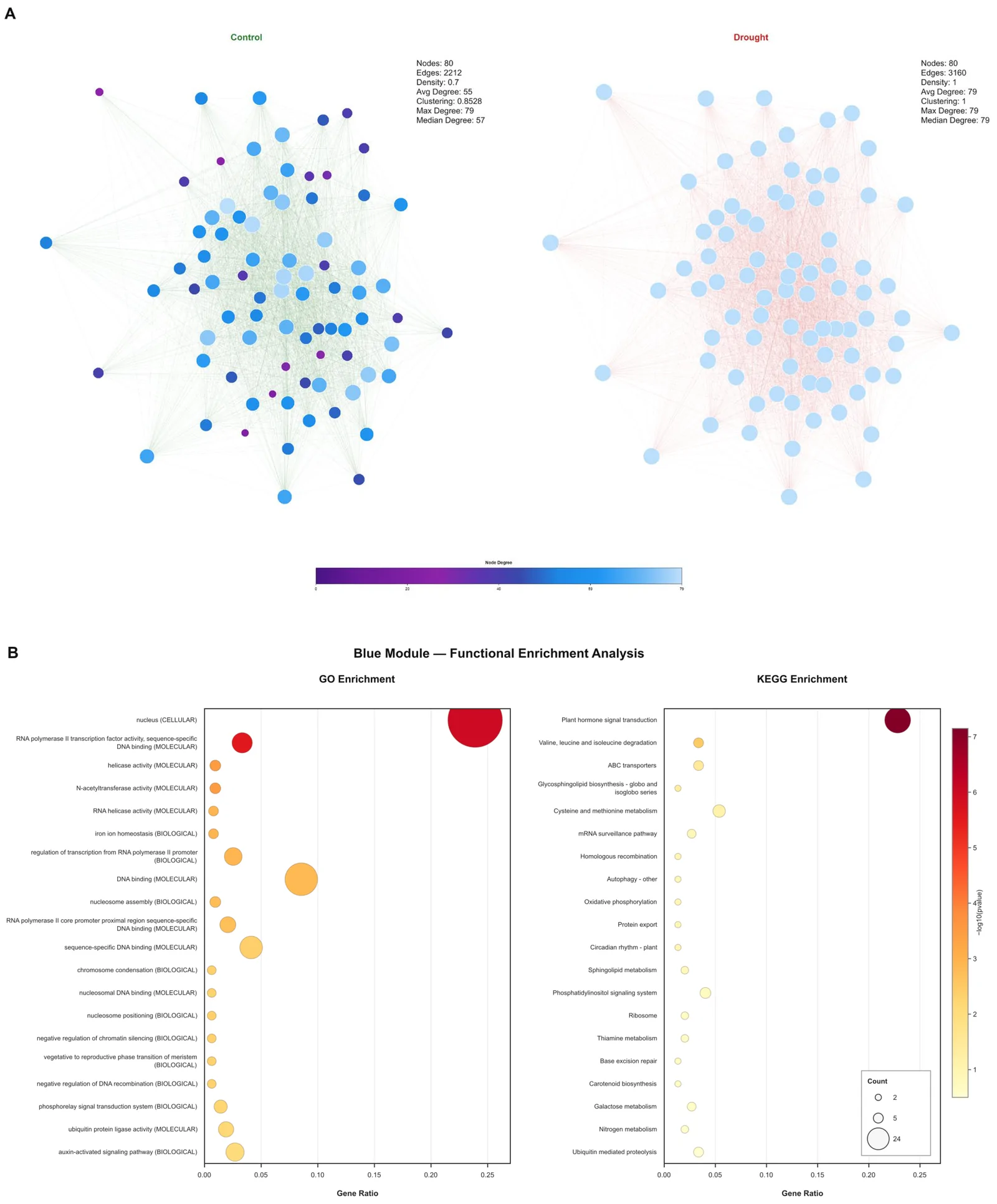
Co-expression network and functional enrichment of the blue module. (**A**) Treatment-specific co-expression networks of the top 80 hub genes in the blue module (*n* = 790 genes) under control (left; *n* = 60 samples) and drought (right; *n* = 60 samples). Node color represents degree, ranging from purple (low) to light blue (high). Edge color indicates treatment: green for the control network (left) and red for the drought network (right). (**B**) Gene Ontology (GO) and KEGG pathway enrichment analysis of the blue module. The bubble plot displays significantly enriched GO terms (hypergeometric test, Benjamini–Hochberg adjusted *p* < 0.05) using genes with 4398 backgrounds. Bubble size represents gene count, and color represents −log_10_(*p*-value).

To examine how the module–trait associations are reflected at the gene-network level, we selected the six modules (blue, brown, turquoise, green, purple, and pink) that were significantly correlated with treatment (*p* < 0.01) from the global WGCNA. We then constructed treatment-specific networks for the same set of genes within each module. For each module, unsigned topological overlap matrices (TOMs) were computed separately from the 60 control and 60 drought samples, using the same soft-thresholding power (β = 8) and a connectivity threshold set at the 75th percentile of TOM values within that module. The 80 genes with the highest total connectivity were selected per module and visualized under both treatments with a shared force-directed layout, enabling direct spatial comparison (Figure 6A; Supplementary Figure S1).

The blue module exhibited the most striking structural transition: its network condensed from a well-connected state (2212 edges, density = 0.70) under control to a near-complete graph under drought (3160 edges, density = 1.00)—a 42.9% edge increase—with all 80 genes remaining in a single fully connected component (Figure 6A,B). Mean topological overlap among the top 80 genes increased by 87.4% (Δ = +0.127). The rewiring was substantial: 2212 edges were retained, while 948 new drought-specific edges emerged (Jaccard index = 0.70), indicating that the blue module transitioned from a moderately connected basal state into a highly integrated stress-responsive configuration. This pattern aligns with the module’s positive correlations with DTI (r = +0.352) and biomass retention traits, suggesting that network tightening under drought contributes to the resistant phenotype despite an overall decline in expression. The other five modules displayed divergent topological responses (Supplementary Figure S1). The brown module severely disintegrated under drought, losing 81.4% of its edges (from 3160 to 587). Conversely, the purple and pink modules, sparse under control conditions, were strongly activated, with edge counts surging by 333.7% and 475.9%, respectively. The turquoise and green modules showed moderate pruning (edge reduction of 21.3% and 16.3%) while maintaining high structural conservation (Jaccard = 0.79 and 0.73).

Functional annotation of the blue module revealed significant enrichment for nuclear localization (151 genes, GeneRatio = 23.9%, *p*_adj_ = 2.94 × 10^−4^), RNA polymerase II transcription factor activity (21 genes, *p*_adj_ = 3.75 × 10^−4^), and the KEGG pathway Plant hormone signal transduction (34 genes, GeneRatio = 22.8%, *p*_adj_ = 3.64 × 10^−6^; Figure 6B), positioning the blue module as a transcription-centered, hormone-responsive regulatory hub. GO and KEGG enrichment analyses were also performed for the brown, turquoise, green, purple, and pink modules (Supplementary Figure S2). The brown module (458 genes) enriches cell wall/extracellular terms and phenylpropanoid biosynthesis, implicating drought-induced wall reinforcement. The green module (268 genes), most strongly enriched, centers on protein folding and chaperone functions (ER protein processing), defining a core proteostasis network. The turquoise module (1203 genes) shows no significant enrichment (*p*_adj_ > 0.05), but top terms (kinase activity, glutathione metabolism, MAPK signaling) suggest phosphorylation and redox regulation. The purple module (85 genes) specifically enriches phosphate-starvation responses and membrane lipid remodeling, pointing to a specialized phosphorus-homeostasis pathway. The pink module (112 genes) links chaperonin-complex, calcium-activated channels, and arginine/proline metabolism to osmolyte synthesis, calcium signaling, and protein quality control.

### 2.5. Interpretable Machine Learning Accurately Predicts Drought Growth Maintenance

**Figure 7:**
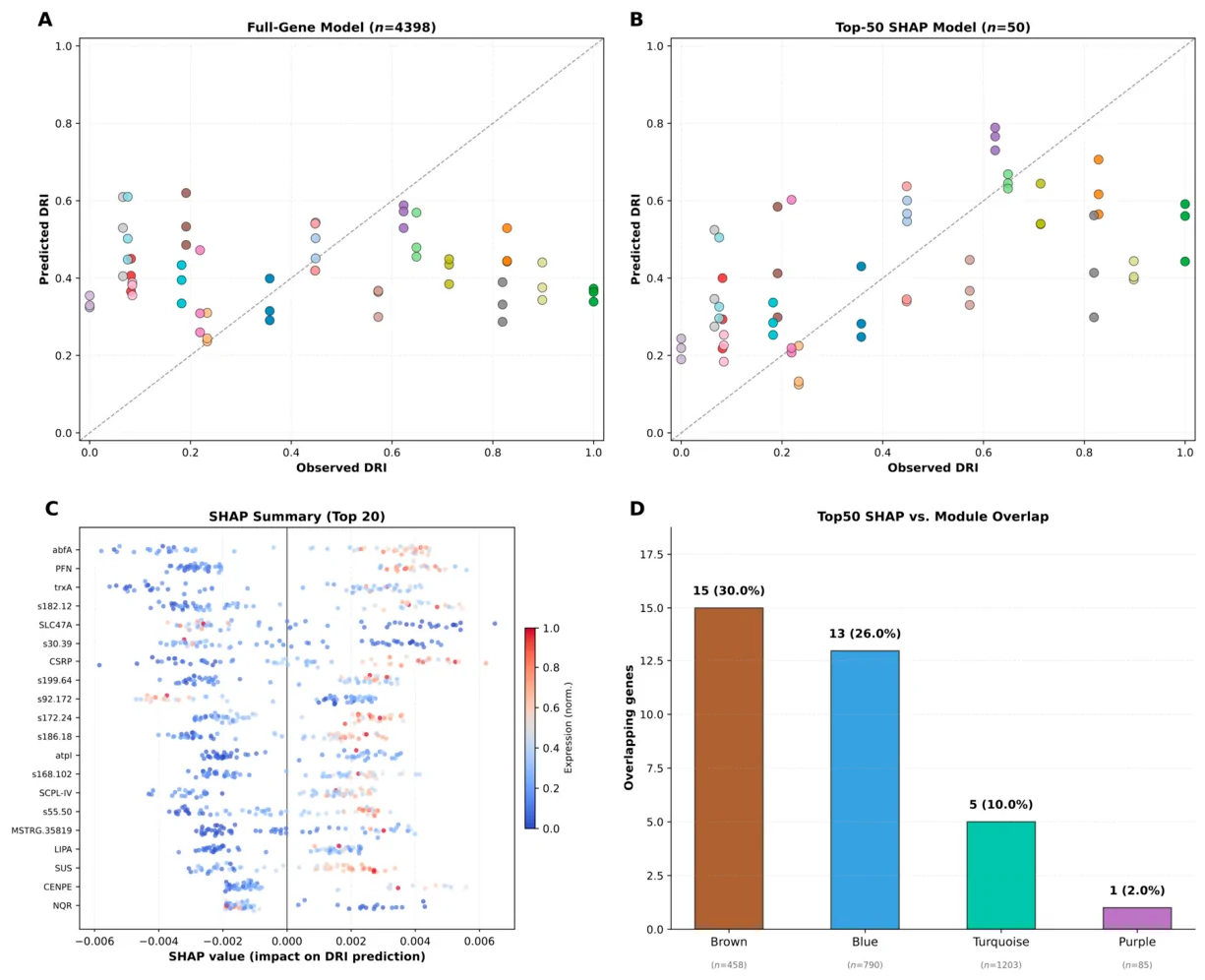
Machine learning prediction of drought resistance index. (**A**,**B**) Leave-one-variety-out cross-validation (LOSOCV) of DTI for 60 drought-stressed samples. Each point represents one sample, colored by variety (*n* = 20). (**A**) Full model using all 4398 genes. Pearson *r* = −0.0153, *p* = 9.08 × 10^−1^, RMSE = 0.3264, R^2^ = −0.1039. (**B**) Reduced model using the top 50 SHAP-ranked genes. Pearson *r* = 0.5886, *p* = 7.58 × 10^−7^, RMSE = 0.2514, R^2^ = 0.3453. (**C**) SHAP beeswarm plot of the top 20 predictive genes. Dots represent samples, colored by expression (blue, low; red, high). SHAP values reflect contribution to DTI prediction; genes are ranked by mean |SHAP|. (**D**) Overlap between the top 50 SHAP genes and four drought-associated WGCNA modules. Bar heights indicate gene counts; module sizes are shown in parentheses.

With the system-level backdrop of transcriptional reorganization established by WGCNA, we next sought to identify which specific genes within these modules most effectively predict genotype-level DTI. To identify a compact gene signature capable of predicting genotype-level drought tolerance, we trained a random forest regression (RFR) model on the 60 drought-stressed samples using all 4398 high-variance genes. Under strict leave-one-variety-out cross-validation (LOSOCV), this full-gene model completely failed to generalize across varieties (Pearson *r* = −0.015, *p* = 0.908; RMSE = 0.326; R^2^ = −0.104; Figure 7A), reflecting severe overfitting driven by the extreme feature-to-sample ratio (4398 genes vs. 57 training samples per fold). We therefore applied TreeSHAP to decompose prediction contributions at the gene level and identified the 50 genes with the highest mean absolute SHAP values. Retraining the RFR model under identical LOSOCV using only these 50 genes dramatically improved prediction: Pearson *r* rose to 0.589 (*p* = 7.58 × 10^−7^), Spearman *ρ* to 0.597 (*p* = 4.87 × 10^−7^), and RMSE dropped to 0.251, with R^2^ reaching 0.345 (Figure 7B). This 88-fold dimensionality reduction not only eliminated overfitting but also confirmed that a compact, interpretable gene set captures the genotype-generalizable component of drought tolerance.

The SHAP beeswarm plot (Figure 7C) revealed the directional contributions of individual predictors: the top-ranked gene *abfA* (ABA signaling) and the transporter *SLC47A* both elevated predicted DTI at high expression, as did *SUS* (sucrose synthase). Other key contributors included *PFN*, *trxA*, *atpI*, and *NQR*, collectively highlighting ABA signaling, redox control, solute transport, carbohydrate metabolism, and energy homeostasis as mechanistic pillars of drought tolerance. Critically, these 50 SHAP-selected genes were not statistical artifacts but converged strongly with the co-expression modules independently associated with drought (Figure 7D). The Brown and Blue modules together accounted for 28 of the 50 genes (56%), the Turquoise module contributed 5, and the Purple module 1. This marked convergence demonstrates that the predictive signature is deeply embedded within the transcriptional network architecture specifically rewired by drought, providing orthogonal validation for both the machine-learning model and the WGCNA-derived module assignments.

### 2.6. Complementary Roles of Lever and Strategy Genes

We next examined the relationship between the SHAP-selected predictive signature and the VAE-decoded strategy-defining genes. Notably, the overlap between the top 50 SHAP genes and the top 50 VAE-decoded genes was minimal—only two genes were shared: CSRP (cysteine-rich protein) and the unannotated L.us.o.g. scaffold9.84. This divergence is not unexpected, as the two methods address fundamentally distinct questions: SHAP prioritizes genes by their marginal predictive power for the continuous DTI, whereas VAE-decoded genes are those that best distinguish the two latent strategy clusters.

Strikingly, despite the minimal gene ID overlap, the two gene sets converged on the same core biological pathways (Table 1). In ABA signaling, SHAP identified abfA (ABA-responsive transcription factor), while VAE identified SNRK2 (central ABA kinase)—upstream regulator vs. downstream effector of the same pathway. In ROS scavenging, SHAP captured trxA and NQR (redox regulators), while VAE captured GST and gpx (direct ROS scavenging enzymes). In protein homeostasis, SHAP identified HSP20 (small heat shock protein), while VAE identified HSPA1s (Hsp70) and VCP (endoplasmic reticulum-associated degradation). In carbon metabolism, SHAP identified SUS (sucrose synthase), while VAE identified AMY (starch degradation) and glnA (glutamine synthesis). This functional stratification reveals that SHAP captures quantitative tuners—genes whose expression level quantitatively scales with DTI—while VAE captures qualitative state switches—genes that define which strategy the system is in, but whose expression may be saturated or binary within each state.

### 2.7. Core Predictive Genes Validation

**Figure 8:**
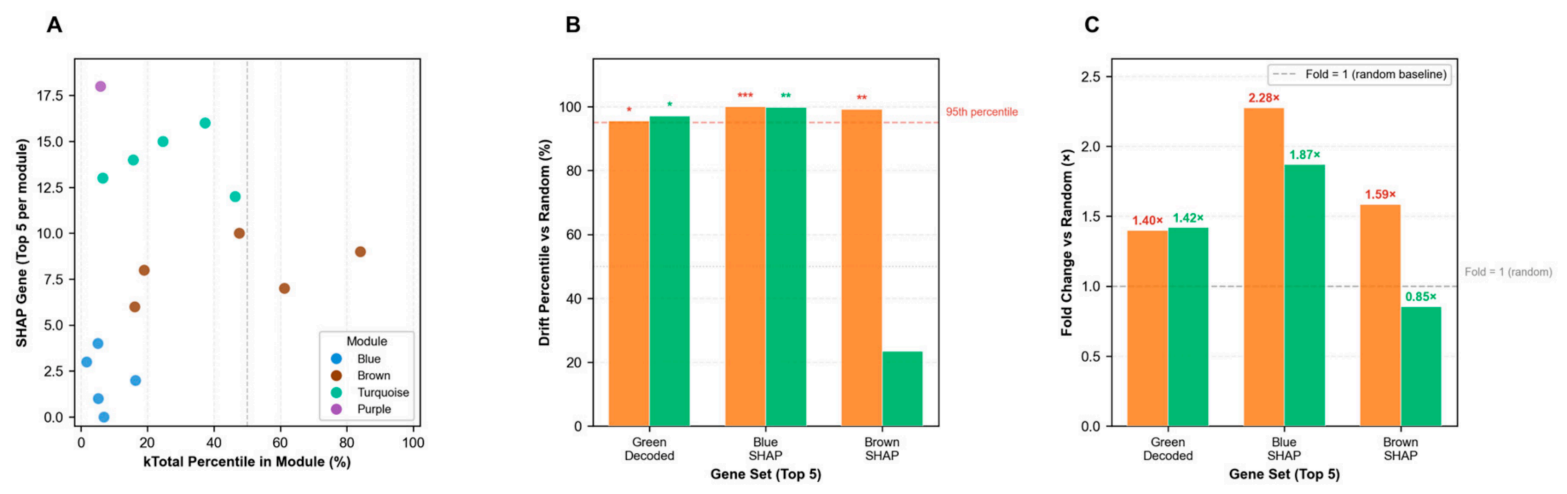
Systematic validation of core predictive genes. (**A**) kTotal percentile of the top 5 SHAP-overlapping genes within each WGCNA module. The dashed line indicates the 50th percentile (median). (**B**) Latent space drift percentile of core gene knockouts relative to 1000 random knockouts, shown separately for Strategy A (growth maintenance, orange) and Strategy B (water conservation, green). (* *p* < 0.05, ** *p* < 0.01, *** *p* < 0.001). The dashed red line marks the 95th percentile. (**C**) Fold change in core gene knockout drift relative to the random knockout mean.

To determine whether the SHAP-identified lever genes occupy central positions within their respective WGCNA modules, we calculated kTotal (intramodular connectivity) for all genes in the four drought-associated modules under drought conditions. For each module, the mean kTotal of the top 5 SHAP-overlapping genes was compared against a 1000-permutation null distribution (Figure 8A). In the Blue module, the mean kTotal of SHAP genes (326.3) fell well below that of random genes (451.3 ± 24.7; *p* < 0.0001, 0th percentile). Similar trends were observed in the Turquoise (503.3 vs. 602.4 ± 62.7; *p* = 0.060, 6th percentile) and purple modules (34.1 vs. 48.6 ± 8.1; *p* = 0.057, 5.7th percentile), while the Brown module showed no significant difference (145.6 vs. 149.0 ± 8.9; *p* = 0.354). Thus, the core predictive genes are consistently not topological hubs; their predictive power does not arise from co-expression centrality.

We next tested whether these non-hub genes nevertheless encode information that the VAE has learned to represent in its compressed latent space. Virtual knockout of three gene sets—VAE-decoded (top 5), Blue-SHAP (top 5), and Brown-SHAP (top 5)—was performed separately for Strategy A (low-DTI, growth maintenance) and Strategy B (high-DTI, water conservation) drought samples. The resulting latent space drift was compared against 1000 random knockouts of equal size (Figure 8B). Five of the six gene set–strategy combinations produced significantly larger drift than random (*p* < 0.05). The Blue-SHAP set caused the largest drift in both strategies, ranking at the 100th percentile in Strategy A (drift = 0.0125 vs. random 0.0055 ± 0.0012; *p* < 0.001) and the 99.8th percentile in Strategy B (drift = 0.0102 vs. random 0.0055 ± 0.0012; *p* = 0.002). The VAE-decoded set was significant in both strategies (Strategy A: 95.5th percentile, *p* = 0.045; Strategy B: 97.1st percentile, *p* = 0.029). Strikingly, the Brown-SHAP set was significant only in Strategy A (99.2nd percentile, *p* = 0.008) but not in Strategy B (23.4th percentile, *p* = 0.766), revealing a strategy-specific dependency.

Across all significant combinations, knockout of core genes induced 1.4–2.3-fold greater latent drift than random knockout (Figure 8C), with the Blue-SHAP set reaching 2.28× in Strategy A. These results confirm that a 4398-dimensional transcriptome compressed into just 8 latent dimensions remains disproportionately sensitive to perturbation of the SHAP-identified lever genes—despite their peripheral network positions. The divergent effect of Brown-SHAP genes between strategies further supports the notion that Strategy A and Strategy B rely on partially distinct molecular programs: Brown module genes, enriched for growth-related processes, are critical for the growth-maintenance strategy (A) but dispensable for the water-conservation strategy (B). By contrast, Blue-SHAP and VAE-decoded genes appear to operate as core components shared across both strategies.

### 2.8. qRT-PCR Validation of Candidate Genes

**Figure 9:**
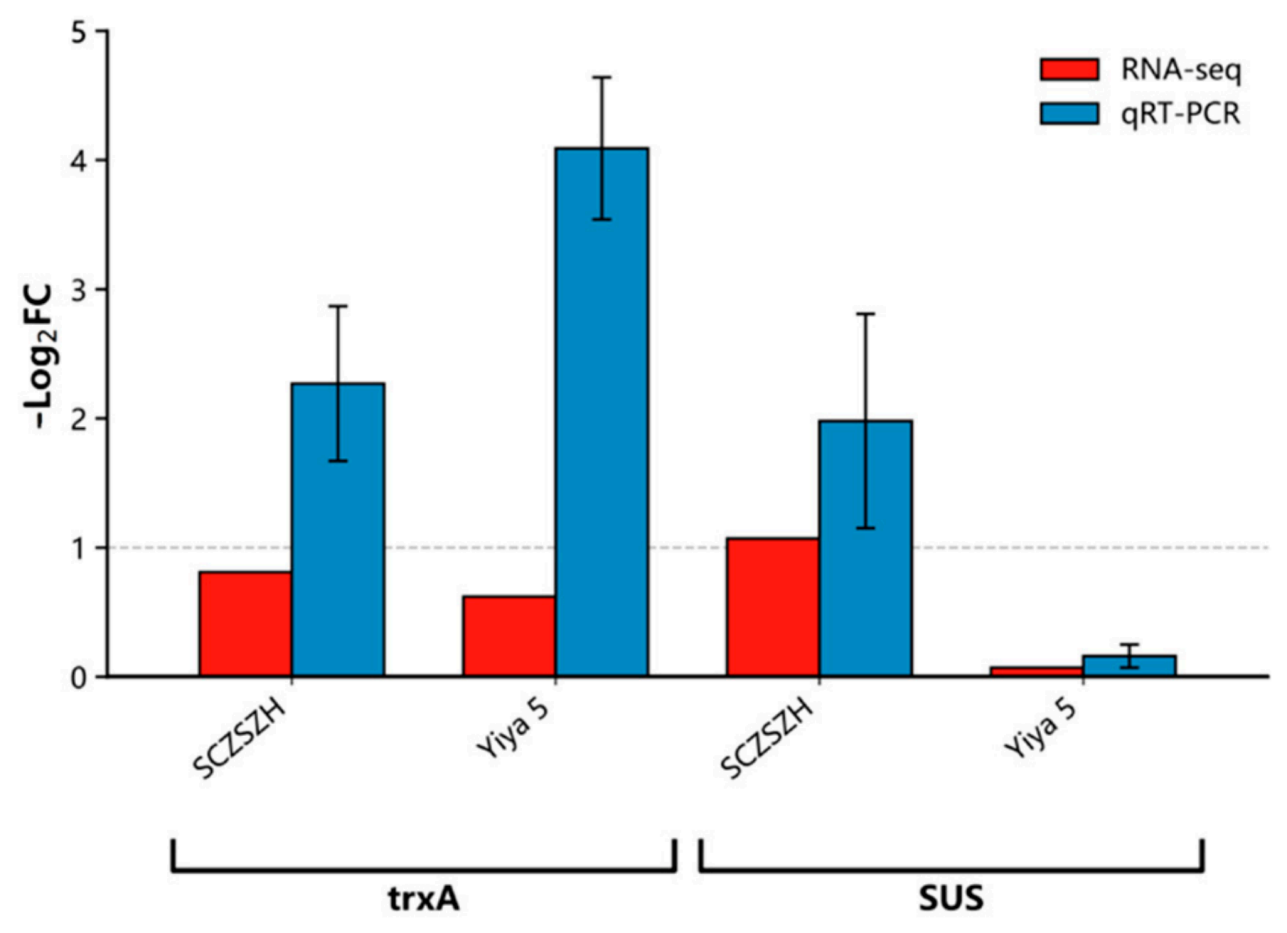
qRT-PCR validation of trxA and SUS in two flax cultivars. Relative expression changes (−Log2FC) of trxA and SUS under drought stress compared to the well-watered control in SCZSZH and Yiya 5. Red bars represent RNA-seq data, and blue bars represent qRT-PCR data. Positive values on the −Log2FC axis indicate down-regulation under drought. Error bars represent the standard error of the mean (SEM) of three biological replicates.

To provide independent technical validation of the transcriptomic data, we performed qRT-PCR on two key SHAP-prioritized genes, trxA and SUS, in two representative cultivars (SCZSZH and Yiya 5). As shown in Figure 9, the qRT-PCR results were highly concordant with the RNA-seq data in terms of the direction of expression change. Both methods consistently showed that trxA and SUS were down-regulated under drought stress across both cultivars (positive values on the −Log2FC axis).

While the qPCR analysis captured a larger magnitude of fold change compared with RNA-seq (e.g., trxA in Yiya 5: qPCR −Log2FC = 4.12 vs. RNA-seq −Log2FC = 0.60), the consistent directional trends strongly validate the reliability of the RNA-seq quantification. Notably, trxA (involved in redox regulation) and SUS (sucrose synthase, involved in carbon metabolism) were among the top 50 SHAP-identified lever genes. Their drought-induced down-regulation is consistent with the drought-repressed pattern of the Blue module (trxA) and the drought-disrupted Brown module (SUS), confirming that the expression decline of these genes is a genuine transcriptional response to drought stress.

## 3. Discussion

Drought stress triggers extensive transcriptional reprogramming in plant roots, orchestrated by complex regulatory networks [26,27,28,29]. However, the extent to which different genotypes within a species share common transcriptional strategies—and how genotypic variation in these strategies translates to phenotypic resilience—remains poorly understood. Here, we used an integrative framework combining β-VAE, WGCNA, and Random Forest–SHAP to examine root transcriptomes of 20 flax varieties with contrasting drought responses. Several aspects of our results merit discussion in the context of existing plant stress biology literature.

Genetic diversity among varieties, shaped by parental background and geographic origin [30,31], often translates into differential phenotypic performance under abiotic stress [32,33,34]. In our study, the 20 flax varieties formed a continuous drought resistance gradient based on DTI, a composite index derived from PCA of biomass and height maintenance ratios, with PC1 (69.0% variance) predominantly loaded by fresh weight and dry weight. This confirms that biomass retention, rather than height maintenance, was the primary dimension of drought resilience in our panel, consistent with studies in spring wheat where higher yields under drought correlated most strongly with total dry matter at maturity [35].

The β-VAE analysis revealed that drought-tolerant and -sensitive varieties differed not in the magnitude of transcriptomic remodeling but in its direction along the latent z_2_ axis. This dichotomy—Strategy A (growth maintenance, low DTI) versus Strategy B (water conservation, high DTI)—echoes the classic “stress tolerance vs. stress avoidance” framework [36] and recent observations in maize roots [37]. That trajectory length did not differ between groups (*p* = 0.604), which aligns with meta-analyses showing that differential expression counts often fail to distinguish tolerant from susceptible genotypes within a species [38,39]. Decoding the latent space revealed functional contrasts consistent with this trade-off: Strategy B invested in proteostasis and detoxification (HSPs, GSTs, SnRK2) [40,41], while Strategy A maintained signatures of active metabolism (laccase, peroxidase, glutamine synthetase). The presence of α-amylase in Strategy B suggests starch mobilization as part of water conservation [42]. This growth-survival trade-off represents a fundamental physiological dilemma, echoing the widely documented “growth-defense trade-off” paradigm [43].

WGCNA revealed that drought triggers functionally selective network reorganization. The blue module—enriched for transcription factors and hormone signaling—paradoxically tightened its internal connectivity under drought (density increasing from 0.70 to 1.00) despite overall expression decline. This “tightening despite down-regulation” suggests a transition from a basal growth-promoting state to a highly coordinated stress-responsive configuration, where remaining hub genes exert stronger regulatory influence per unit expression [44]. This phenomenon of “quantitative reduction but qualitative intensification” has been observed in stress-responsive networks in Arabidopsis and rice, where key transcription factors show enhanced connectivity under stress while expression levels remain stable or decrease [45,46,47]. By contrast, growth-related modules (brown) disintegrated, while specialized stress modules (purple, pink) were activated—reflecting adaptive prioritization of survival over growth. A particularly illuminating finding is the minimal overlap between SHAP-identified lever genes and VAE-decoded strategy markers (only 2 out of 50), yet their convergence on common pathways—ABA signaling, ROS scavenging, protein homeostasis, and carbohydrate metabolism. This reveals a functional stratification: SHAP captures quantitative “tuners” (*abfA*, *SUS*) whose expression scales continuously with DTI, while VAE captures qualitative “switches” (*SNRK2*, *AMY*) that define strategy orientation. This tuner-switch model has practical implications: tuners offer targets for incremental improvement within a given strategy, while switches determine fundamental strategic orientation and may be more relevant for introducing novel resistance mechanisms across germplasm.

Perhaps the most counterintuitive finding is that SHAP-identified lever genes are consistently not network hubs (Blue module SHAP genes at the 0th percentile of kTotal). This challenges the assumption that centrality equates to importance. We propose these genes function as levers—nodes with low connectivity but critical positions in information flow. Virtual knockout further supported their potential functional importance: removal of five blue-SHAP genes induced 2.28-fold greater latent drift than random knockout. This distinction has practical implications: hub manipulation may generate pleiotropic effects, while lever genes may offer more specific and predictable outcomes, expanding the toolkit of candidate markers beyond traditional hub-centric selection.

The use of in silico gene perturbation to investigate gene function has been demonstrated in previous studies, including transcription factor perturbation in Arabidopsis thaliana [48] and virtual knockout analyses in animal systems, where computational predictions successfully recapitulated major findings from real knockout experiments [49]. These studies provide precedent for using computational perturbation approaches to assess the potential functional importance of candidate genes, supporting the application of virtual knockout in our study. Virtual knockout further revealed strategy-specific dependencies: brown-SHAP genes (growth-related) were critical only in Strategy A, not Strategy B, indicating that the two adaptation programs rely on partially distinct molecular infrastructure. By contrast, blue-SHAP and VAE-decoded genes were significant in both strategies, suggesting a “shared backbone + strategy-specific modules” architecture. This hierarchy resembles immune systems, where innate immunity provides universal defense while adaptive immunity tailors specific responses [50]. Breeding could thus target both levels: strengthening the shared backbone for basal improvement, or modulating strategy-specific modules to customize adaptation to target environments [51,52].

The biological relevance of our computational framework is supported by the assignment of drought-associated genes to specific co-expression modules and transcriptional strategies. The ABA-responsive transcription factor gene *abfA* was assigned to the Blue module, which was enriched for transcription factor activity and plant hormone signal transduction and was positively correlated with DTI; *abfA* is involved in ABA-mediated drought signaling and ABA-responsive gene expression [53]. The thioredoxin-related gene *trxA* was also assigned to the Blue module, consistent with a role in redox regulation during abiotic stress. In contrast, the sucrose synthase gene *SUS* was assigned to the Brown module, which was disrupted under drought, linking stress-related carbon metabolism to drought adaptation. The SNRK2 family gene and the starch-degradation gene *AMY* were identified as VAE-decoded Strategy B genes, consistent with the roles of SNRK2 in ABA signaling and AMY in carbohydrate metabolism [54,55]. The enrichment of the Blue module in plant hormone signaling and transcription factor-associated functions is also consistent with established plant stress-response networks [56,57]. Collectively, these findings support the biological plausibility of our computational predictions. However, the specific functions of the identified flax candidates require further experimental validation. Several limitations should be acknowledged. Our transcriptomic data are from a single time point and tissue; drought responses are dynamic, and time-series and multi-tissue analyses would provide a more complete picture [58,59]. DTI was based on shoot traits while transcriptomics focused on roots; direct root architectural measurements would strengthen phenotype-genotype linkage. The 20-variety panel, while informative, may lack power for subtle effects; larger panels and integration with QTL mapping or GWAS would enhance validation [60,61]. Functional assignments rely on homology-based annotation; experimental validation via gene editing is needed to confirm causal roles. While the in silico virtual knockout approach provides a powerful way to quantify the non-redundant information of candidate genes, it is a computational approximation and may not fully capture the complex in vivo regulatory dynamics. Nevertheless, the validity of our perturbation framework is supported by the concordance between RNA-seq and our qRT-PCR results for key lever genes (e.g., *trxA* and *SUS*), as well as the established application of in silico knockouts in other plant systems to recapitulate biological outcomes. Future experimental validation using CRISPR/Cas9-based gene editing in flax is necessary to confirm the causal roles of these candidate genes and to translate these computational predictions into molecular breeding applications.

Despite these caveats, our integrative framework demonstrates that combining dimensionality reduction, network analysis, and interpretable machine learning can uncover hierarchical and complementary regulatory layers invisible to any single method. The identification of tuners and switches, hubs and levers, and shared and strategy-specific modules provides a nuanced view of drought resistance architecture that transcends the traditional binary classification of tolerant versus susceptible genotypes. This framework is adaptable to other stress types and crop species, enabling systematic mapping of genotype-to-phenotype relationships at the systems level.

## 4. Materials and Methods

### 4.1. Plant Materials and Drought Treatment

A total of 20 flax (*Linum usitatissimum* L.) cultivars/lines were used in this study, comprising 18 drought-tolerant and 2 drought-sensitive genotypes, as listed in Table 2. The field experiment was conducted at the experimental station of the Inner Mongolia Academy of Agricultural and Animal Husbandry Sciences (Inner Mongolia, China) starting on 30 April 2022. The experimental field is characterized by a Brown earth soil type with a loam texture. The physical and chemical properties of the topsoil (0–20 cm layer) were as follows: soil organic matter 17.53 g/kg, total nitrogen 0.83 g/kg, total phosphorus 0.53 g/kg, total potassium 17.90 g/kg, alkali-hydrolyzable nitrogen 53.21 mg/kg, available phosphorus 25.70 mg/kg, and available potassium 113.60 mg/kg. The average soil bulk density was 1.33 g/cm^3^. A split-plot design was employed, with water regime as the main-plot factor (two levels) and cultivar as the sub-plot factor (20 levels). Each cultivar × treatment combination was replicated three times in individual plots of 9 m^2^. Plots were separated by 0.5 m buffer rows and surrounded by a 2 m-wide protective border.

Prior to sowing, the field was deeply plowed, cleared of large rocks, and harrowed flat. After a baseline flood irrigation, the plots were marked, and seeds were manually sown in shallow furrows at a fixed sowing rate. All plots were maintained under natural conditions until the full-bloom stage was reached across all genotypes. A mobile rainout shelter was then erected to strictly control water supply, and two irrigation treatments were imposed: (1) control (CK), in which soil water content was maintained at 70–75% of field capacity; and (2) severe drought (SD), in which soil water content was maintained at 25–30% of field capacity. Soil moisture was monitored every two days using a TDR soil moisture meter (TDR 300, Spectrum Technologies, Aurora, IL, USA) combined with the gravimetric oven-drying method (using a five-point sampling strategy per plot for the 0–20 cm layer). When soil water content approached the lower limits of the target range, manual sprinkler irrigation was applied. Soil moisture was re-measured the following day to prevent exceeding the target thresholds. To rule out the effects of lateral water movement and surface runoff, a supplementary pot experiment (using identical cultivars and water treatments) was conducted in parallel within the rainout shelter. All phenotypic data (plant height, shoot fresh weight, and shoot dry weight) reported in this study represent the mean values derived from both the field and pot experiments.

After 30 days of continuous treatment, when 80% of leaves of the two drought-sensitive cultivars (Neiya No. 9 and Longza No. 1) showed severe wilting or lodging, and approximately 50% of the drought-tolerant cultivars displayed varying degrees of wilting, plants were harvested simultaneously across all plots. The shoots were excised and immediately used for phenotypic measurements. The entire root systems were then carefully excavated, rinsed thoroughly with deionized water, blotted dry, and immediately snap-frozen in liquid nitrogen. Root samples were stored at −80 °C until RNA extraction.

### 4.2. Phenotypic Measurements and Calculation of the Drought Tolerance Index (DTI)

For each plot, plant height (cm), shoot fresh weight (g), and shoot dry weight (g) were measured on the three sampled plants. Plant height was determined as the absolute distance from the stem base to the growing point using a tape measure. Shoot fresh weight was determined immediately after harvest using an analytical balance (precision: 0.1 mg). For dry weight determination, shoots were first oven-dried at 105 °C for 30 min to inactivate enzymes, and then dried at 65 °C to constant weight. The plot-level value was taken as the mean of the three individual plants.

To quantitatively assess the drought tolerance of each cultivar, three growth maintenance ratios were calculated based on the plot means:

Shoot dry weight maintenance ratio = mean dry weight under SD/mean dry weight under CK.

Shoot fresh weight maintenance ratio = mean fresh weight under SD/mean fresh weight under CK.

Plant height maintenance ratio = mean plant height under SD/mean plant height under CK.

For each cultivar and treatment, the plot-level values were first averaged across the three replicate plots to obtain a single mean value for shoot dry weight, shoot fresh weight, and plant height. The maintenance ratio for each trait was then calculated as the ratio of the mean under SD to the mean under CK, yielding one value per cultivar for each ratio. These ratios were then subjected to Z-score standardization across the 20 cultivars [value = (ratio − mean)/SD]. Principal component analysis (PCA) was performed on the standardized ratios using the 20 cultivars as observations and the three ratios as variables. The first principal component (PC1) scores were extracted and defined as the Drought Tolerance Index (DTI). For ease of interpretation and visualization, the DTI values were linearly rescaled to the 0–1 interval: DTI_scaled = (DTI − DTI_min)/(DTI_max − DTI_min). The resulting scaled DTI was used as the phenotypic response variable in all downstream association analyses and machine learning modeling.

### 4.3. RNA Extraction, Library Construction, Sequencing, and Data Availability

Total RNA was extracted from the root samples using the TRIzol-based method (Invitrogen). Genomic DNA was removed by DNase I treatment (TaKaRa, Kyoto, Japan). RNA quality was assessed with a NanoDrop 2000 (Thermo Fisher Scientific, Wilmington, DE, USA) and Agilent 2100 Bioanalyzer(Agilent, Waldbronn, Germany); only samples with OD260/280 = 1.8–2.2, OD260/230 ≥ 2.0, RIN ≥ 6.5, and 28S:18S ≥ 1.0 were used. Sequencing libraries were constructed with the TruSeq RNA Sample Preparation Kit (Illumina, San Diego, CA, USA) and sequenced on an Illumina NovaSeq 6000 platform (paired-end 150 bp). Raw reads were filtered with fastp v0.19.5 (quality score <20 at 5′ end, <3 at 3′ end, length <30 bp discarded). The raw transcriptome data are available in the NCBI Sequence Read Archive (SRA) under BioProject PRJNA1046211.

### 4.4. Transcriptome Data Preprocessing

Raw reads after quality filtering were mapped to the flax reference genome (GCA_010665275.2) [61] using HISAT2 (v2.1.0) [62] with default parameters. Gene-level read counts were obtained using featureCounts (v2.0.1) [63], and transcripts per million (TPM) values [64] were calculated from the counts. A total of 120 RNA-seq libraries (20 cultivars × 2 treatments × 3 biological replicates) were included in the analysis.

For all downstream analyses, genes were filtered in two steps: (i) only genes with TPM > 1 in all 120 samples were retained (17,593 genes); (ii) after log2(TPM + 1) transformation, the coefficient of variation (CV = standard deviation/mean) was calculated for each gene across the 120 samples. The top 25% of genes with the highest CV were selected, yielding 4398 high-variability genes. No differential expression *p*-values or FDR thresholds were used for this filtering step; the selection was based entirely on expression variance to capture the most dynamically regulated transcriptional signals.

### 4.5. PCA, UMAP, and β-VAE Latent Space Analysis

PCA was performed on the standardized 4398-gene matrix using scikit-learn(v1.3.2) [65]. For cultivar-level visualization, mean expression per variety × treatment was computed across biological replicates, and PCA and UMAP [66] were applied to the resulting 40 centroids. UMAP was run with default parameters (n_neighbors = 15, min_dist = 0.1).

A β-VAE [67] implemented using PyTorch (v1.13.0). The encoder and decoder each comprised two hidden layers (512 and 256 neurons) with LayerNorm and LeakyReLU activations. The model was trained for 300 epochs using the Adam optimizer (learning rate = 1 × 10^−3^, weight decay = 1 × 10^−5^, batch size = 32) with a mean squared error reconstruction loss and a ReduceLROnPlateau scheduler. The latent space dimension was set to 8, and the KL divergence was weighted by β = 4.0.

Hyperparameters were selected based on a grid search over β ∈ {1, 2, 4, 6, 8, 10} and latent dimensions d ∈ {2, 4, 8, 16, 32}. For each combination, we evaluated reconstruction loss (MSE) on the training set and the absolute Pearson correlation between the resulting z_2_ coordinates and DTI. β = 4 was chosen because it balanced reconstruction fidelity and latent disentanglement, consistent with previous β-VAE studies. β ≥ 6 led to posterior collapse (KL divergence → 0), while β = 1 resulted in highly entangled latent dimensions that failed to separate treatment groups. d = 8 was selected as the minimum dimension that stably separated Control from Drought samples while avoiding overfitting; d ≥ 16 led to non-convergent KL loss given the sample size (*n* = 120). Training used the Adam optimizer (learning rate = 1 × 10^−3^, weight decay = 1 × 10^−5^) for 300 epochs with mean squared error reconstruction loss and a ReduceLROnPlateau scheduler (patience = 30, factor = 0.5). Loss converged from 315.0 at epoch 100 to 111.4 at epoch 300, indicating stable training.

To extract a biologically interpretable latent subspace, we performed Partial Least Squares (PLS) regression on the 60 drought-treated samples, using the full 8-dimensional latent representation Z ∈ ℝ^60×8^ as predictors and DTI as the response variable, with two PLS components extracted. The first PLS component (PLS1) captured the dominant latent direction aligned with DTI variation, and its Pearson correlation with DTI was calculated to quantify the strength of the latent-to-phenotype association. For each variety, adaptation trajectories were defined as the vector from the Control centroid to the Drought centroid in the (z_1_, z_2_) space. K-means clustering (k = 2) on the drought-centroid z_2_ coordinates classified varieties into two strategies. Gene drivers of strategy divergence were identified by decoding the mean expression profiles of the two strategy groups and selecting the top 50 genes by absolute log_2_ fold-change.

### 4.6. Weighted Gene Co-Expression Network Analysis

In this study, WGCNA was performed using highly variable genes selected based on their coefficient of variation (CV) across all 120 samples, rather than differential expression significance (*p*-values or FDR). This strategy prioritizes genes with substantial expression variability for identifying coordinated transcriptional patterns and is consistent with approaches adopted in previous studies [68,69].

A signed weighted co-expression network was built using the WGCNA R package (v1.72-1) [21]. A soft-threshold power β = 8 was selected to achieve a scale-free topology fit R^2^ > 0.80. The adjacency matrix was computed as |cor|^β^, and the topological overlap matrix (TOM) was derived. Modules were identified by average-linkage hierarchical clustering of 1 − TOM, using a dynamic tree cut algorithm with a minimum module size of 30 genes and a cut height of 0.25 for merging modules with eigengene dissimilarity < 0.15. Module eigengenes (MEs) were defined as the first principal component of each module’s expression matrix.

Pearson correlations between MEs and traits—treatment (drought vs. control), DTI, fresh weight retention, dry weight retention, and plant height retention—were calculated. *p*-values were Bonferroni-corrected for 35 tests (7 modules × 5 traits). For each trait-associated module, hub genes were identified as the top 30 genes ranked by |gene significance| × |module membership|.

For each module, GO Biological Process and KEGG pathway enrichment was performed using Fisher’s exact test against the 4398-gene background. The Benjamini–Hochberg FDR was applied for multiple testing correction; for modules with extreme sizes, both raw and adjusted *p*-values were considered.

To assess drought-induced structural reorganization of co-expression modules, we constructed treatment-specific networks for the six modules that exhibited significant correlations with Treatment (*p* < 0.01). For each module, unsigned topological overlap matrices (TOMs) were computed separately using the 60 control and 60 drought samples, applying the soft-thresholding power β = 8 determined from the WGCNA. A hard threshold was imposed at the 75th percentile of TOM values within each module to retain only robust connections. The 80 genes with the highest total intramodular connectivity were selected from each module for network visualization. Networks were rendered with a force-directed layout shared between control and drought panels to enable direct topological comparison. Node color was mapped to degree (purple, low; light blue, high). All network properties—edge count, density, average degree, clustering coefficient, average path length, and number of connected components—were calculated using the igraph R package.

### 4.7. Random Forest Regression and SHAP Interpretability Analysis

To predict the drought tolerance index from transcriptomic profiles, the 60 drought-treated samples (20 cultivars × 3 replicates) were used as the modeling population. The feature set comprised the 4398 highly variable genes, and the target variable was DTI_scaled. A Random Forest regressor [70] was built with scikit-learn v1.3.2, using the following hyperparameters: n_estimators = 500, max_features = ‘sqrt’, random_state = 42, and all other parameters at their default values.

To control overfitting and assess generalizability, a leave-one-genotype-out cross-validation (LOCO-CV) strategy was adopted. In each of the 20 iterations, all three replicates of one cultivar were held out as the test set, while the remaining 19 cultivars (57 samples) were used for training. After completing the 20 iterations, the predicted DTI values for all test samples were pooled, and the Pearson correlation coefficient (r) between observed and predicted DTI, as well as the RMSE, were calculated.

Subsequently, a final model was trained on the entire set of drought-treated samples and subjected to SHAP analysis. We applied the Tree SHAP algorithm implemented in the shap package (v0.42.1) to compute the marginal contribution of each gene to the prediction. Genes were ranked in descending order of their mean absolute SHAP values, and the top 50 were designated as the “core predictive gene set”. To validate the predictive capacity of this gene set, we re-executed the LOCO-CV regression using only these 50 genes as features and compared the resulting performance with that of the full model.

### 4.8. Virtual Knockout and Latent Space Drift

To assess whether the core predictive genes identified by SHAP [71] analysis occupy topologically central positions within co-expression modules, we computed the total connectivity (kTotal, defined as the sum of absolute Pearson correlation coefficients between a given gene and all other genes within the same module) for every gene in the four significant modules (Blue, Brown, Turquoise, Purple) using expression data from the 60 drought-treated samples. For each module, the mean kTotal of the top 5 SHAP-overlapping genes was compared against a null distribution generated by 1000 random samplings of equal-sized gene sets from the same module. One-sided permutation *p* values were calculated as the proportion of random samples with mean kTotal less than or equal to the observed SHAP gene mean.

To evaluate whether the core predictive genes carry non-redundant information in the latent transcriptomic space, a β-variational autoencoder (β = 4, 8 latent dimensions, 4398 input genes) was retrained on the full 120-sample expression matrix (log_2_-transformed and standardized). Three gene sets were tested: (i) the top 5 VAE-decoded genes, (ii) the top 5 SHAP genes overlapping with the blue module, and (iii) the top 5 SHAP genes overlapping with the Brown module. Virtual knockout was performed by setting the expression of target genes to zero (i.e., the mean after standardization) for drought samples only. The Euclidean distance between pre- and post-knockout latent coordinates was averaged across target samples to quantify latent space drift. Knockout was performed separately for Strategy A (growth maintenance, *n* = 27) and Strategy B (water conservation, *n* = 33) drought samples. For each gene set–strategy combination, a null distribution was generated by 1000 random gene knockouts of equal size, and one-sided permutation *p* values were computed as the proportion of random knockouts with drift greater than or equal to the observed value.

### 4.9. Statistical Analysis

All statistical tests were performed using R 4.4.1 or Python 3.10. Unless otherwise specified, the data are expressed as mean ± standard error (SEM). Single-factor ANOVA and Tukey HSD post hoc tests are used for multi-group comparisons, and two-tailed *t*-tests are used for two-group comparisons. A *p*-value < 0.05 was regarded as a significant difference.

### 4.10. qRT-PCR Validation

qRT-PCR was performed using the same total RNA to validate the RNA-seq data. Reactions were run on an Applied Biosystems 7500 Real-Time PCR System (Life Technologies, Carlsbad, CA, USA) with TB Green Premix Ex Taq II (TaKaRa). Each 20 μL reaction contained 10 μL of 2 × TB Green Premix Ex Taq II, 0.5 μL of each primer (10 μM), 0.4 μL of ROX Reference Dye (50×), 1 μL of diluted cDNA, and 7.6 μL of ddH_2_O. The amplification conditions were: 95 °C for 30 s; 40 cycles of 95 °C for 5 s and 60 °C for 34 s, followed by melting curve analysis. The gene L.us.o.g.scaffold155.126 (annotated in the reference genome GCA_010665275.2) was used as the internal reference gene. Relative expression was calculated using the 2^−ΔΔCt^ method with three biological replicates per cultivar–treatment combination. Primer sequences are listed in Supplementary Table S3.

## 5. Conclusions

Increasingly, integrative computational frameworks that combine unsupervised learning, network analysis, and interpretable machine learning are being applied to dissect complex stress responses in crops. We found that, while PCA and WGCNA successfully captured the dominant treatment effects and module-level transcriptional reorganization, the β-VAE latent space modeling and SHAP-based feature attribution were able to uncover strategy-level divergence and non-hub predictive genes that were not identifiable through conventional approaches. Integration of these methods revealed that drought resilience is determined by the direction—rather than the magnitude—of transcriptomic reprogramming, and that peripheral lever genes, rather than network hubs, carry non-redundant predictive information. Identification of two distinct adaptation strategies (water-conserving vs. growth-maintenance) and their associated gene sets (e.g., *SNRK2*, *AMY*, *HSPA1s* for Strategy B; laccase, peroxidase, *glnA* for Strategy A), along with 50 SHAP-identified lever genes (*abfA*, *PFN*, *trxA*, *SLC47A*, *SUS*), may provide biologically meaningful targets for breeding. With further development, this framework may also be adapted for other stress types and other crop species, enabling systematic mapping of genotype-to-phenotype relationships at the systems level.

## Figures and Tables

**Table 1:** Functional complementarity of SHAP and VAE gene sets within shared biological pathways.

Pathway	SHAP Gene (Lever/Tuner)	VAE Gene (State/Switch)	Regulatory Relationship
ABA signaling	abfA (transcription factor)	SNRK2 (kinase)	Upstream kinase → downstream TF
ROS scavenging	trxA, NQR (redox regulators)	GST, gpx (scavenging enzymes)	Redox regulation → direct scavenging
Protein homeostasis	HSP20 (sHSP)	HSPA1s (Hsp70), VCP	Primary protection → folding/degradation
Carbon metabolism	SUS (sucrose synthase)	AMY (starch degradation)	Sucrose utilization → starch mobilization
Cytoskeleton	PFN (profilin)	CML, KATNA1 (calcium/ATPase)	Actin binding → calcium-mediated regulation

**Table 2:** Information on flax (*Linum usitatissimum* L.) cultivars used in this study.

No.	Cultivar Name	Origin (Province/Country)	Drought Tolerance Type
1	Longya No. 8	Gansu, China	Drought-Tolerant
2	Dingya No. 15	Gansu, China	Drought-Tolerant
3	Dingxi No. 17	Gansu, China	Drought-Tolerant
4	Lixian	Gansu, China	Drought-Tolerant
5	Gaolanbai	Gansu, China	Drought-Tolerant
6	Longya 10	Gansu, China	Drought-Tolerant
7	Zhangya No. 1	Hebei, China	Drought-Tolerant
8	Zhangya No. 2	Hebei, China	Drought-Tolerant
9	Baxuan No. 3	Hebei, China	Drought-Tolerant
10	Baya No. 15	Hebei, China	Drought-Tolerant
11	Ningya No. 15	Ningxia, China	Drought-Tolerant
12	Ningya No. 17	Ningxia, China	Drought-Tolerant
13	Ningya No. 19	Ningxia, China	Drought-Tolerant
14	Yiya No. 4	Xinjiang, China	Drought-Tolerant
15	Shache zaoshuzhonghong	Xinjiang, China	Drought-Tolerant
16	Yiya No. 5	Xinjiang, China	Drought-Tolerant
17	R43	Inner Mongolia, China	Drought-Tolerant
18	BGOLDXREDWING44 × 3	USA	Drought-Tolerant
19	Neiya No. 9	Inner Mongolia, China	Drought-Sensitive
20	Longza No. 1	Gansu, China	Drought-Sensitive

## Data Availability

The transcriptome (RNA-seq) data used in this study were deposited in the Sequence Read Archive (SRA) of the NCBI database (BioProject ID: PRJNA1046211).

## Supplementary Materials

The following supporting information can be downloaded at: https://www.mdpi.com/article/10.3390/plants15213494/s1. Supplementary Figure S1. Treatment-specific co-expression networks of the brown, turquoise, green, purple, and pink modules under control and drought conditions. Supplementary Figure S2. GO and KEGG enrichment analyses of the brown, turquoise, green, purple, and pink modules. Supplementary Table S1. Top50 VAE-decoded genes differentially expressed between Strategy A (growth maintenance) and Strategy B (water conservation). Supplementary Table S2. GO and KEGG Enrichment of Strategy A&B. Supplementary Table S3. Primers used for qRT-PCR.
