# Supplementary material for: Latent-Space Modeling Reveals Transcriptional Strategy Divergence in Flax Root Drought Response
Source: Plants (Basel). 2026 Nov 9;15(21):3494. doi: 10.3390/plants15213494 (PMC13766076; doi:10.3390/plants15213494)
Supplement: Supplementary file 1 [file plants-15-03494-s001.zip › Figure S1-S2.pdf]

Figure S1. Treatment-specific co-expression networks of the brown, turquoise, green, purple, and pink modules. For each module, the top 80 hub genes are visualized under control (left) and drought (right) conditions, using the same layout, coloring scheme, and thresholding criteria as in Figure 5. The 75th-percentile TOM thresholds for each module are: brown = 0.132, turquoise = 0.213, green = 0.246, purple = 0.087, pink = 0.133.

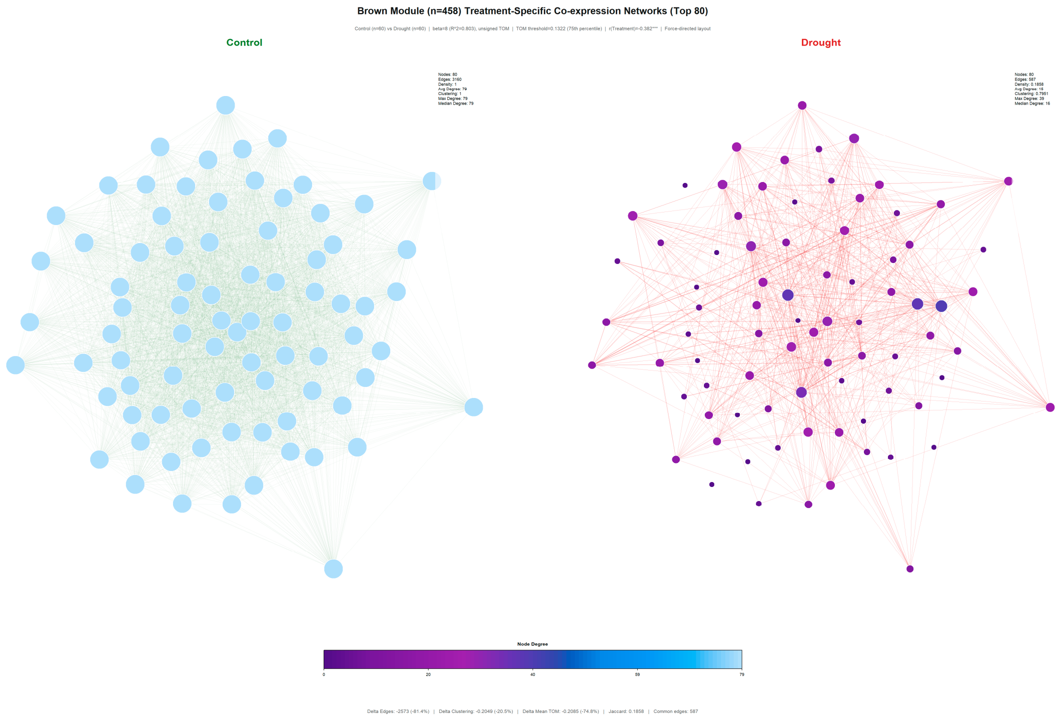

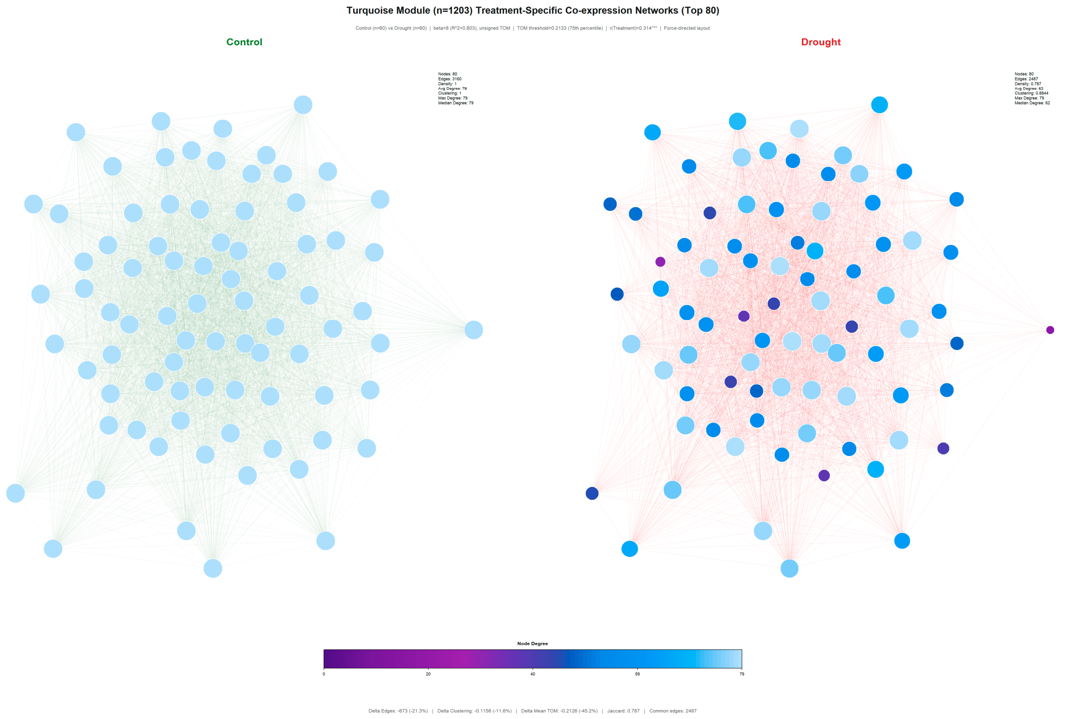
Turquoise Module (n=1203) Treatment-Specific Co-expression Networks (Top 80)
Control (n=60) vs Drought (n=60) | beta=8 (R^2=0.803), unsigned TOM | TOM threshold=0.2133 (75th percentile) | r(Treatment)=0.314*** | Force-directed layout
Control
Nodes: 80
Edges: 3160
Density: 1
Avg Degree: 79
Clustering: 1
Max Degree: 79
Median Degree: 79
Drought
Nodes: 80
Edges: 2487
Density: 0.787
Avg Degree: 62
Clustering: 0.8844
Max Degree: 79
Median Degree: 62
Node Degree
0
20
40
59
79
Delta Edges: -673 (-21.3%) | Delta Clustering: -0.1156 (-11.6%) | Delta Mean TOM: -0.2126 (-45.2%) | Jaccard: 0.787 | Common edges: 2487

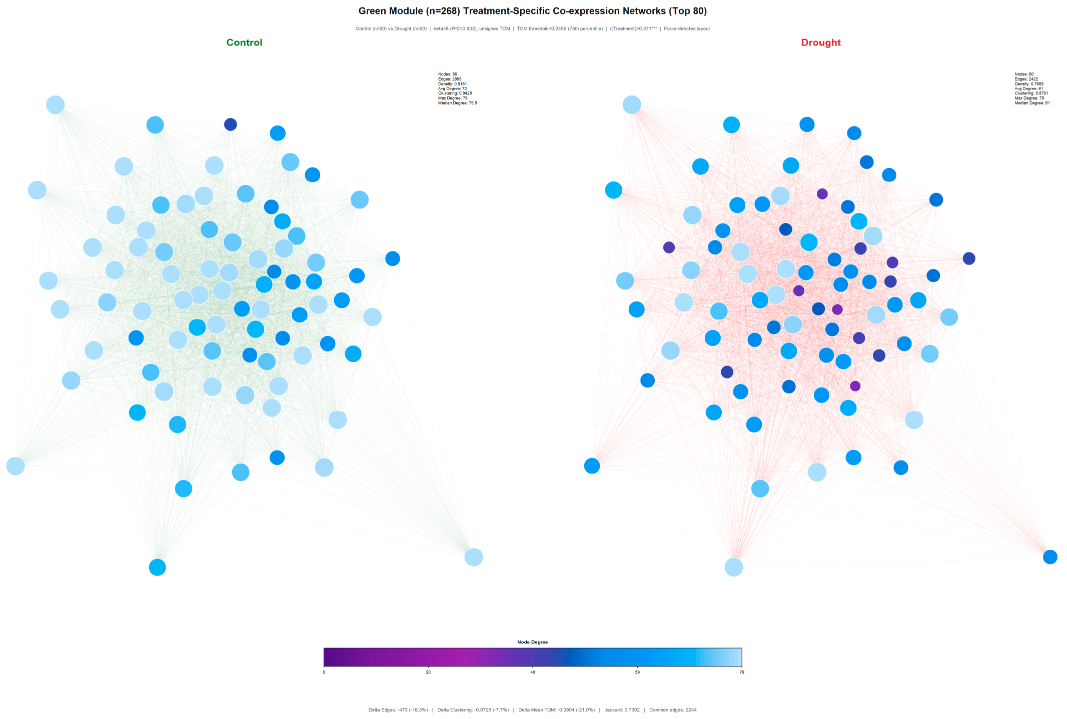
Green Module (n=268) Treatment-Specific Co-expression Networks (Top 80)
Control (n=60) vs Drought (n=60) | beta=8 (R^2=0.803), unsigned TOM | TOM threshold=0.2459 (75th percentile) | r(Treatment)=0.371*** | Force-directed layout
Control
Nodes: 80
Edges: 2895
Density: 0.9161
Avg Degree: 72
Clustering: 0.9428
Max Degree: 79
Median Degree: 75.5
Drought
Nodes: 80
Edges: 2422
Density: 0.7665
Avg Degree: 61
Clustering: 0.8701
Max Degree: 79
Median Degree: 61
Node Degree
0
20
40
59
79
Delta Edges: -473 (-16.3%) | Delta Clustering: -0.0726 (-7.7%) | Delta Mean TOM: -0.0804 (-21.0%) | Jaccard: 0.7302 | Common edges: 2244

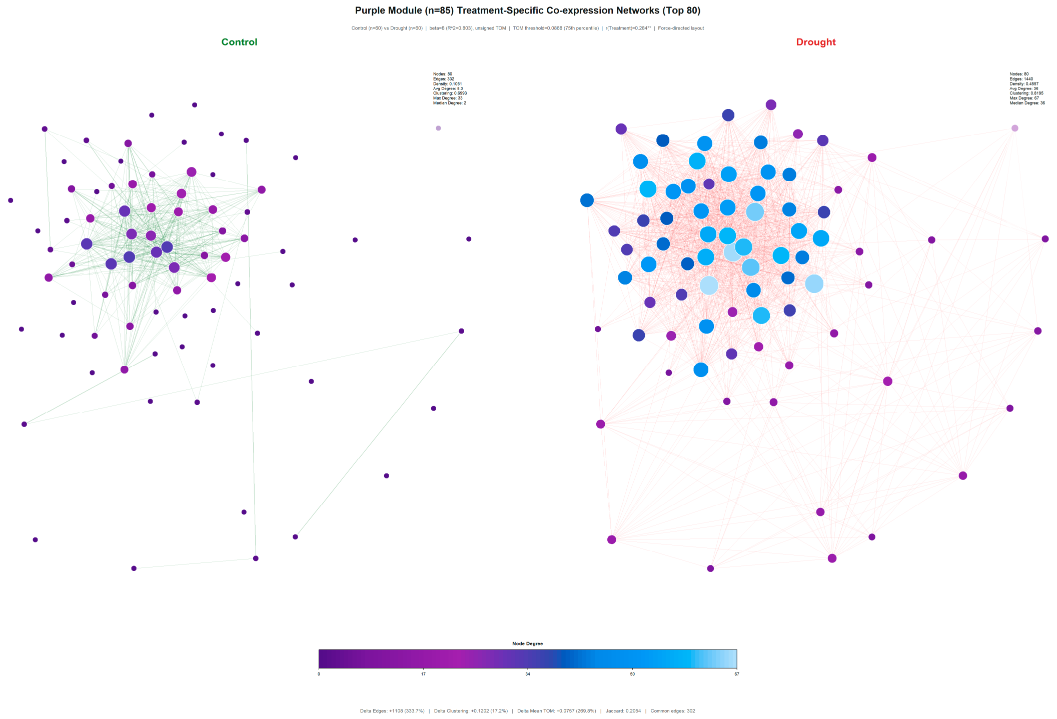
Purple Module (n=85) Treatment-Specific Co-expression Networks (Top 80)
Control (n=60) vs Drought (n=60) | beta=8 (R^2=0.803), unsigned TOM | TOM threshold=0.0868 (75th percentile) | r(Treatment)=0.284** | Force-directed layout
Control
Drought
Nodes: 80
Edges: 332
Density: 0.1051
Avg Degree: 8.3
Clustering: 0.6993
Max Degree: 33
Median Degree: 2
Nodes: 80
Edges: 1440
Density: 0.4557
Avg Degree: 36
Clustering: 0.8195
Max Degree: 67
Median Degree: 36
Node Degree
0
17
34
50
67
Delta Edges: +1108 (333.7%) | Delta Clustering: +0.1202 (17.2%) | Delta Mean TOM: +0.0757 (269.8%) | Jaccard: 0.2054 | Common edges: 302

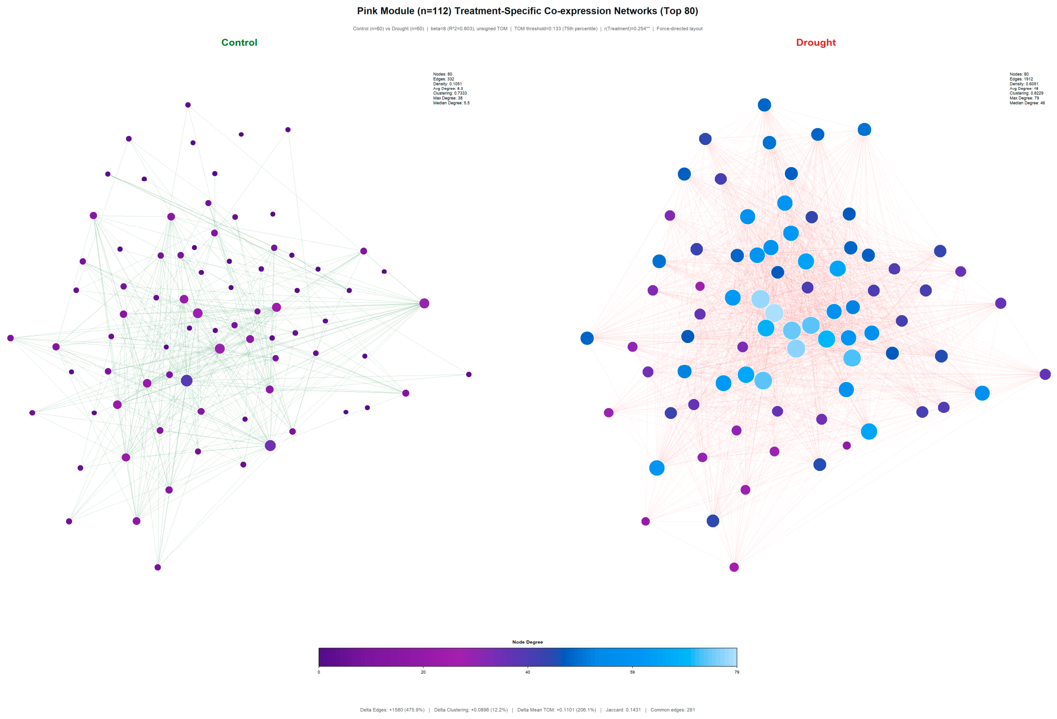
Pink Module (n=112) Treatment-Specific Co-expression Networks (Top 80)
Control (n=60) vs Drought (n=60) | beta=8 (R^2=0.803), unsigned TOM | TOM threshold=0.133 (75th percentile) | r(Treatment)=0.254** | Force-directed layout
Control
Drought
Nodes: 80
Edges: 332
Density: 0.1051
Avg Degree: 8.3
Clustering: 0.7333
Max Degree: 38
Median Degree: 5.5
Nodes: 80
Edges: 1912
Density: 0.6051
Avg Degree: 48
Clustering: 0.8229
Max Degree: 79
Median Degree: 46
Node Degree
0
20
40
59
79
Delta Edges: +1580 (475.9%) | Delta Clustering: +0.0896 (12.2%) | Delta Mean TOM: +0.1101 (206.1%) | Jaccard: 0.1431 | Common edges: 281

Figure S2. Functional enrichment analysis of WGCNA modules associated with drought treatment. brown, turquoise, green, purple, and pink modules.

Bubble plots showing GO Biological Process (left) and KEGG pathway (right) enrichment results for each drought-responsive co-expression module. Bubble size represents the number of module genes annotated to each term; color intensity indicates statistical significance as -log10(p-value).

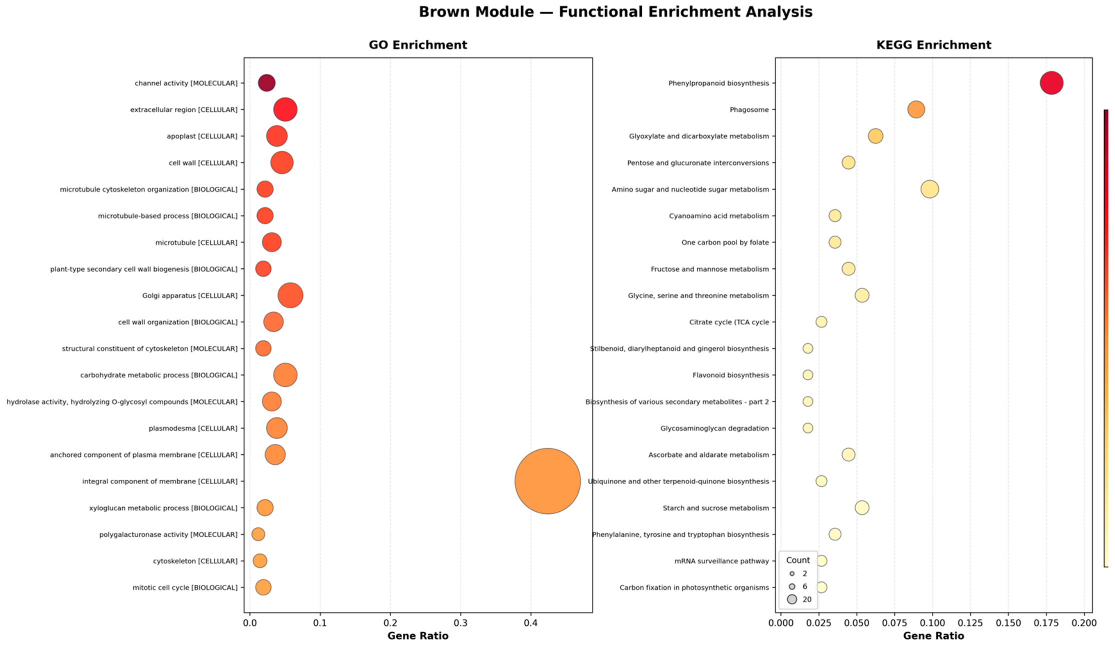

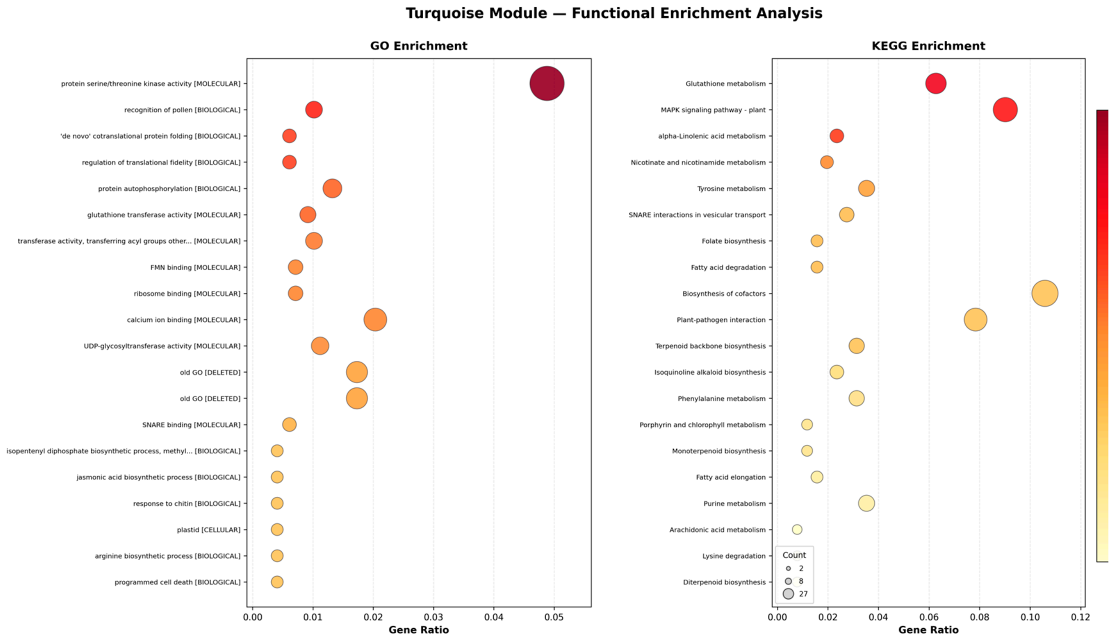

Turquoise Module — Functional Enrichment Analysis
GO Enrichment
protein serine/threonine kinase activity [MOLECULAR]
recognition of pollen [BIOLOGICAL]
'de novo' cotranslational protein folding [BIOLOGICAL]
regulation of translational fidelity [BIOLOGICAL]
protein autophosphorylation [BIOLOGICAL]
glutathione transferase activity [MOLECULAR]
transferase activity, transferring acyl groups other... [MOLECULAR]
FMN binding [MOLECULAR]
ribosome binding [MOLECULAR]
calcium ion binding [MOLECULAR]
UDP-glycosyltransferase activity [MOLECULAR]
old GO [DELETED]
old GO [DELETED]
SNARE binding [MOLECULAR]
isopentenyl diphosphate biosynthetic process, methyl... [BIOLOGICAL]
jasmonic acid biosynthetic process [BIOLOGICAL]
response to chitin [BIOLOGICAL]
plastid [CELLULAR]
arginine biosynthetic process [BIOLOGICAL]
programmed cell death [BIOLOGICAL]
0.00
0.01
0.02
0.03
0.04
0.05
Gene Ratio
KEGG Enrichment
Glutathione metabolism
MAPK signaling pathway - plant
alpha-Linolenic acid metabolism
Nicotinate and nicotinamide metabolism
Tyrosine metabolism
SNARE interactions in vesicular transport
Folate biosynthesis
Fatty acid degradation
Biosynthesis of cofactors
Plant-pathogen interaction
Terpenoid backbone biosynthesis
Isoquinoline alkaloid biosynthesis
Phenylalanine metabolism
Porphyrin and chlorophyll metabolism
Monoterpenoid biosynthesis
Fatty acid elongation
Purine metabolism
Arachidonic acid metabolism
Lysine degradation
Diterpenoid biosynthesis
Count
2
8
27
0.00
0.02
0.04
0.06
0.08
0.10
0.12
Gene Ratio

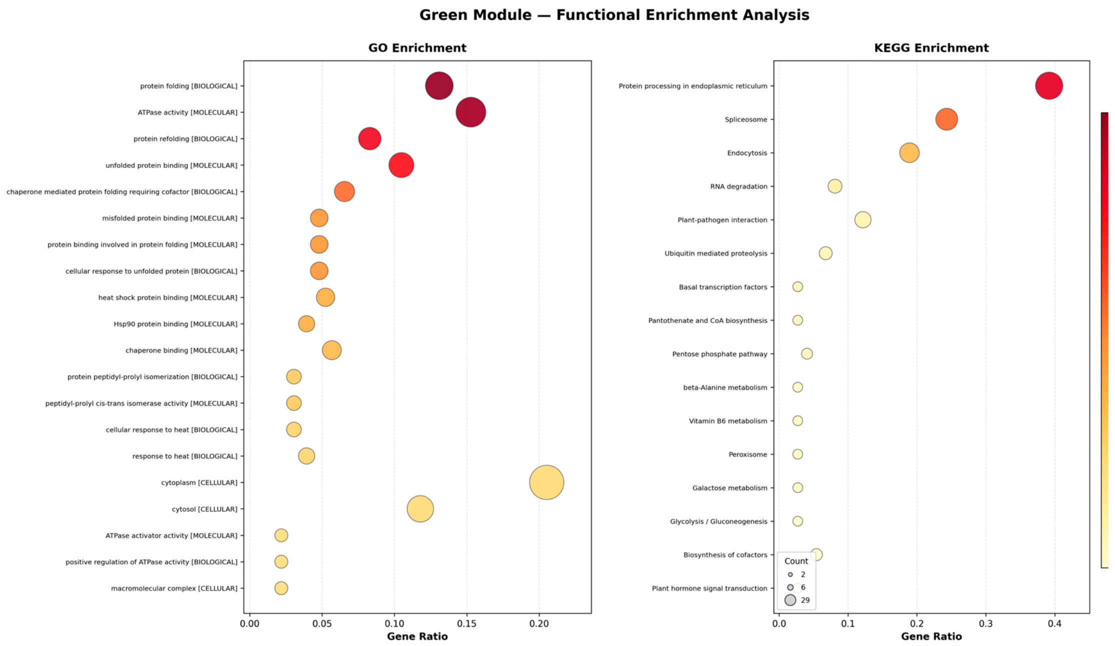

Green Module — Functional Enrichment Analysis
GO Enrichment
protein folding [BIOLOGICAL]
ATPase activity [MOLECULAR]
protein refolding [BIOLOGICAL]
unfolded protein binding [MOLECULAR]
chaperone mediated protein folding requiring cofactor [BIOLOGICAL]
misfolded protein binding [MOLECULAR]
protein binding involved in protein folding [MOLECULAR]
cellular response to unfolded protein [BIOLOGICAL]
heat shock protein binding [MOLECULAR]
Hsp90 protein binding [MOLECULAR]
chaperone binding [MOLECULAR]
protein peptidyl-prolyl isomerization [BIOLOGICAL]
peptidyl-prolyl cis-trans isomerase activity [MOLECULAR]
cellular response to heat [BIOLOGICAL]
response to heat [BIOLOGICAL]
cytoplasm [CELLULAR]
cytosol [CELLULAR]
ATPase activator activity [MOLECULAR]
positive regulation of ATPase activity [BIOLOGICAL]
macromolecular complex [CELLULAR]
0.00
0.05
0.10
0.15
0.20
Gene Ratio
KEGG Enrichment
Protein processing in endoplasmic reticulum
Spliceosome
Endocytosis
RNA degradation
Plant-pathogen interaction
Ubiquitin mediated proteolysis
Basal transcription factors
Pantothenate and CoA biosynthesis
Pentose phosphate pathway
beta-Alanine metabolism
Vitamin B6 metabolism
Peroxisome
Galactose metabolism
Glycolysis / Gluconeogenesis
Biosynthesis of cofactors
Plant hormone signal transduction
Count
2
6
29
0.0
0.1
0.2
0.3
0.4
Gene Ratio

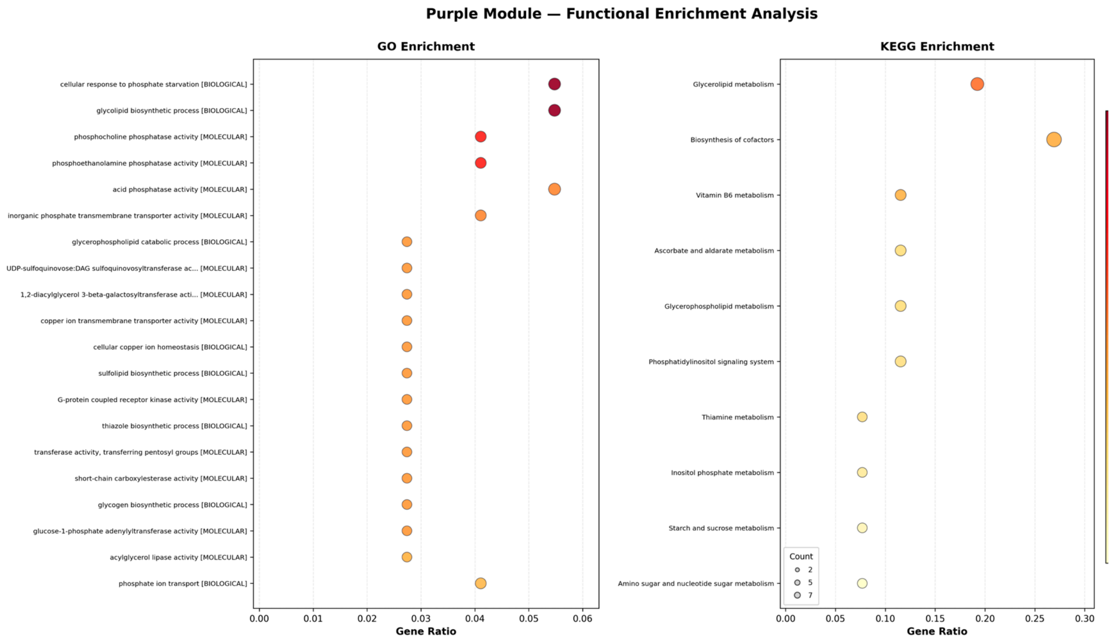
Purple Module — Functional Enrichment Analysis
GO Enrichment
cellular response to phosphate starvation [BIOLOGICAL]
glycolipid biosynthetic process [BIOLOGICAL]
phosphocholine phosphatase activity [MOLECULAR]
phosphoethanolamine phosphatase activity [MOLECULAR]
acid phosphatase activity [MOLECULAR]
inorganic phosphate transmembrane transporter activity [MOLECULAR]
glycerophospholipid catabolic process [BIOLOGICAL]
UDP-sulfoquinovose:DAG sulfoquinovosyltransferase ac... [MOLECULAR]
1,2-diacylglycerol 3-beta-galactosyltransferase acti... [MOLECULAR]
copper ion transmembrane transporter activity [MOLECULAR]
cellular copper ion homeostasis [BIOLOGICAL]
sulfolipid biosynthetic process [BIOLOGICAL]
G-protein coupled receptor kinase activity [MOLECULAR]
thiazole biosynthetic process [BIOLOGICAL]
transferase activity, transferring pentosyl groups [MOLECULAR]
short-chain carboxylesterase activity [MOLECULAR]
glycogen biosynthetic process [BIOLOGICAL]
glucose-1-phosphate adenylyltransferase activity [MOLECULAR]
acylglycerol lipase activity [MOLECULAR]
phosphate ion transport [BIOLOGICAL]
0.00
0.01
0.02
0.03
0.04
0.05
0.06
Gene Ratio
KEGG Enrichment
Glycerolipid metabolism
Biosynthesis of cofactors
Vitamin B6 metabolism
Ascorbate and aldarate metabolism
Glycerophospholipid metabolism
Phosphatidylinositol signaling system
Thiamine metabolism
Inositol phosphate metabolism
Starch and sucrose metabolism
Amino sugar and nucleotide sugar metabolism
Count
2
5
7
0.00
0.05
0.10
0.15
0.20
0.25
0.30
Gene Ratio

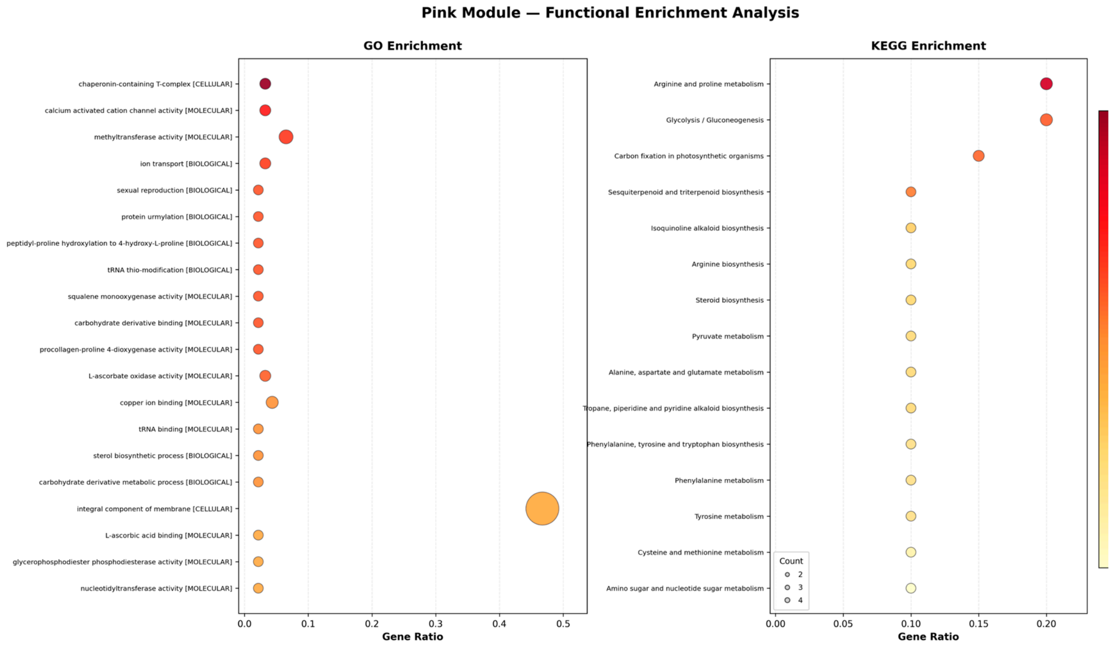
Pink Module — Functional Enrichment Analysis
GO Enrichment
chaperonin-containing T-complex [CELLULAR]
calcium activated cation channel activity [MOLECULAR]
methyltransferase activity [MOLECULAR]
ion transport [BIOLOGICAL]
sexual reproduction [BIOLOGICAL]
protein urmylation [BIOLOGICAL]
peptidyl-proline hydroxylation to 4-hydroxy-L-proline [BIOLOGICAL]
tRNA thio-modification [BIOLOGICAL]
squalene monooxygenase activity [MOLECULAR]
carbohydrate derivative binding [MOLECULAR]
procollagen-proline 4-dioxygenase activity [MOLECULAR]
L-ascorbate oxidase activity [MOLECULAR]
copper ion binding [MOLECULAR]
tRNA binding [MOLECULAR]
sterol biosynthetic process [BIOLOGICAL]
carbohydrate derivative metabolic process [BIOLOGICAL]
integral component of membrane [CELLULAR]
L-ascorbic acid binding [MOLECULAR]
glycerophosphodiester phosphodiesterase activity [MOLECULAR]
nucleotidyltransferase activity [MOLECULAR]
0.0
0.1
0.2
0.3
0.4
0.5
Gene Ratio
KEGG Enrichment
Arginine and proline metabolism
Glycolysis / Gluconeogenesis
Carbon fixation in photosynthetic organisms
Sesquiterpenoid and triterpenoid biosynthesis
Isoquinoline alkaloid biosynthesis
Arginine biosynthesis
Steroid biosynthesis
Pyruvate metabolism
Alanine, aspartate and glutamate metabolism
Tropane, piperidine and pyridine alkaloid biosynthesis
Phenylalanine, tyrosine and tryptophan biosynthesis
Phenylalanine metabolism
Tyrosine metabolism
Cysteine and methionine metabolism
Amino sugar and nucleotide sugar metabolism
Count
2
3
4
0.00
0.05
0.10
0.15
0.20
Gene Ratio
